# Design, evaluation, cytotoxic activity, molecular docking, ADMET analysis, and dynamic simulations and the preparation of new isoxazoles, thiazoles, 1,3-thiazines, and thiazolopyrimidines derived from quinoline-pyridopyrimidines

**DOI:** 10.1080/13880209.2025.2547744

**Published:** 2025-08-19

**Authors:** Ameen A. Abu-Hashem, Nasser Amri, Ahmed F. El-Sayed

**Affiliations:** ^a^ Department of Physical Sciences, Chemistry Division, College of Science, Jazan University, Jazan, Saudi Arabia; ^b^ Photochemistry Department (Heterocyclic Unit), National Research Centre, Dokki, Egypt; ^c^ Microbial Genetics Department, Biotechnology Research Institute, National Research Centre, Giza, Egypt; ^d^ Egypt Center for Research and Regenerative Medicine (ECRRM), Cairo, Egypt

**Keywords:** Quinoline, thiazole, isoxazole, thiazine, pyrido[2,3-d] pyrimidine, pyridothiazolo-pyrimidine, anticancer activity, molecular docking, ADMET, dynamic simulations

## Abstract

**Context:**

Quinoline, isoxazole, and pyridothiazolopyrimidinone derivatives are novel compounds with significant biological activity, exhibiting anticancer properties and holding promising therapeutic applications.

**Objective:**

This investigation synthesized new heterocyclic compounds in high yields from quinoline-2-thioxo-pyridopyrimidinone and assessed their anticancer activities. Additionally, it conducted molecular docking, ADMET analysis, and molecular dynamics simulations.

**Materials and methods:**

A new series of quinoline-pyridothiazolopyrimidine derivatives has been synthesized using advanced techniques. The structures of the new compounds were confirmed using IR, NMR, MS and elemental analysis. All compounds were tested *in vitro* for their anticancer activity.

**Results:**

Isoxazole and thiazolopyridopyrimidinones displayed the highest activity against several cancer cell lines. Docking simulations revealed that compounds **5d**, **5e**, **11a**, and **11b** exhibited favorable binding energies and effectively interacted with the active sites of the EGFR, CDK2, ERα, and VEGFR receptors. The ADMET analysis of these compounds demonstrated compliance with Pfizer’s rules. Molecular dynamics simulations confirmed the stability of complexes formed by compounds **5d**, **11a**, and **11b** with CDK2, ERα, VEGFR, and EGFR. The root mean square deviation (RMSD) values were recorded, while the root mean square fluctuation (RMSF) values ranged from 0.10 to 0.6 nm. The solvent-accessible surface area (SASA) values were measured to be between 135–145 nm^2^, 125–130 nm^2^, 155–165 nm^2^, and 160–175 nm^2^.

**Discussion and conclusions:**

The cytotoxicity (IC_50_) and selectivity index are presented in Tables. Molecular docking analyses showed that compounds **5d**, **5e**, **11a**, and **11b** demonstrated significant binding energies. These consistent results support the notion that both practical and theoretical studies align regarding the anticancer properties of these new compounds. Furthermore, these findings emphasize the potential of these compounds in ongoing drug development efforts.

## Introduction

**Figure 1.**
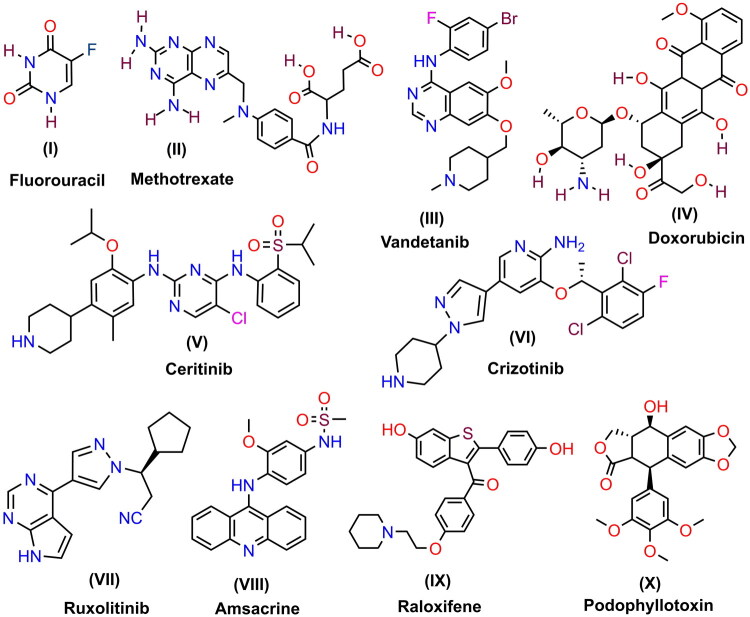
The chemical structures of various drugs used as anticancer treatments.

Cancer is a disease characterized by the uncontrolled growth of specific cells in the body, which can spread to other areas. It can originate in almost any part of the human body, consisting of trillions of cells. Human cells grow and multiply through cell division, producing new cells as needed. When cells become too large or damaged, they die, allowing new cells to form. However, this orderly process can sometimes go awry, leading to the growth and multiplication of abnormal or damaged cells. These cells can form tumors, which are masses of tissue. Tumors can be either cancerous or noncancerous (benign). Cancerous tumors invade nearby tissues and can also travel to distant parts of the body to create new tumors, known as metastases. Cancerous tumors are often referred to as malignant tumors. Cancer is one of the leading causes of death worldwide. In 2022, there were approximately 20 million new cases of cancer and 9.7 million cancer-related deaths globally. It is projected that the number of new cancer cases will rise to 29.9 million by 2040, with cancer-related deaths increasing to 15.3 million. Generally, cancer rates are highest in countries with greater life expectancy, higher education levels, and better living standards. Recent developments reflect significant progress in both basic and clinical cancer research, with translational research emerging as a key paradigm. New approaches to Personalized cancer Medicine and Targeted Therapies have shown promising advancements. The most common cancers, listed in descending order of expected new cases in 2024, are breast cancer, prostate cancer, lung and bronchus cancer, colorectal cancer, melanoma, bladder cancer, kidney and renal pelvis cancer, lymphoma, endometrial cancer, ovarian cancer, pancreatic cancer, leukemia, thyroid cancer, and liver cancer, [National Cancer Institute, USA, Bobst International Center 160 East 53rd Street, New York, NY 10022] Doctors and researchers have identified various practical methods for combating cancer. One of the most commonly used treatments is chemotherapy, which utilizes powerful chemicals to target and eliminate rapidly growing cells in the body. This approach is efficient for cancer patients, as cancer cells grow and reproduce much faster than normal cells. Consequently, chemotherapy is often the preferred treatment option for many cancer patients. Numerous chemotherapy drugs are available, each with unique properties and mechanisms of action. Some of these include Fluorouracil **(I)**, Methotrexate **(II)**, Vandetanib **(III)**, Doxorubicin **(IV)**, Ceritinib **(V)**, Crizotinib **(VI)**, Ruxolitinib **(VII)**, Amsacrine **(VIII)**, Raloxifene **(IX)**, Podophyllotoxin **(X**), as illustrated in Figure 1.

**Figure 2.**
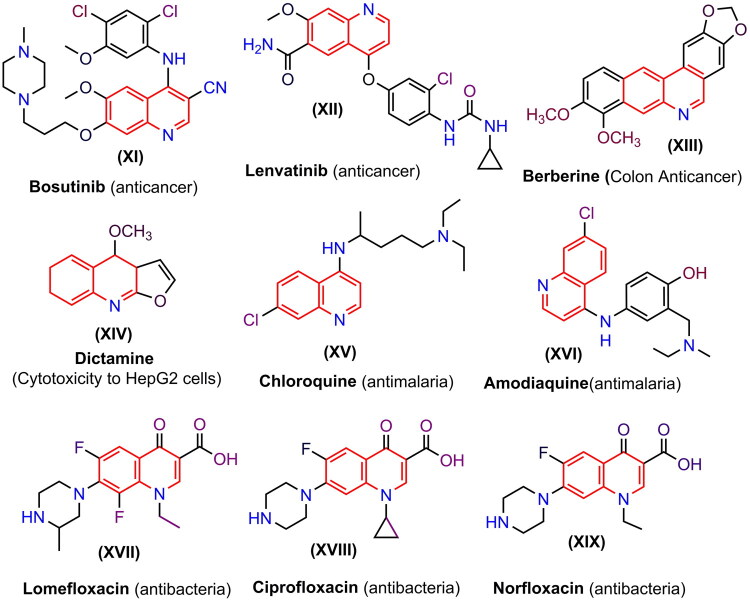
The chemical structures of different drugs derived from quinoline.

A literature survey has shown that pyrido[2,3-*d*] pyrimidine derivatives possess significant pharmaceutical and medicinal chemistry potential due to their diverse biological activities. As a result, they form an essential structural component in many drugs. These derivatives exhibit various biological and pharmacological effects, including anti-inflammatory properties (El-Gazzar et al. 2009; Dasari et al. 2020; Rani and Kunta 2021). Antimicrobial (Abdelhameed et al. 2020; Elsaedany et al. 2021; Ibrahim and Allehyani 2021). Antioxidant (Dangolani et al. 2018; Gouda et al. 2020; Khajouei et al. 2021). Antiviral (DeGoey et al. 2013). Antitubercular (Rajesh et al. 2011). Also used to inhibit certain enzymes, including glucosidase (Panahi et al. 2013; Adib et al. 2018). Adenosine kinase (Zheng et al. 2003). Threonine tyrosine kinase (TTK) (Huang et al. 2021). And dihydrofolate reductase (Gangjee et al. 2001; Wang et al. 2017). Heterocyclic compounds have a diverse range of physical, chemical, and biological properties due to the presence of multiple rings, including pyrimidines, pyrazoles, thiophenes, furans, thiazoles, and others (Mahmoud et al. 2013; Hashem et al. 2017; Ramadan and El-Helw 2018; Ramadan et al. 2021; Elgubbi et al. 2024). One notable compound, Thieno[2,3-*d*] pyrimidine derivatives, exhibits various biological activities, including antitumor, anticancer, and antioxidant effects (Abu-Hashem et al. 2010, 2020, 2021a; Abu-Hashem and Hussein 2015; Abu-Hashem and Al-Hussain 2022). Besides, pyrazolopyrimidines, pyrimidoquinoxalines, pyrrolothiazolo­pyrimidines, triazolo-pyrrolothiazolopyramidines, thiazolopyrimidines, pyrimidoquinazolines and azolopyrimido-­quinolines possess various beneficial properties, including cytotoxic, anticancer, antitumor, antioxidant, analgesic, and anti-inflammatory effects (El-Gazzar et al. 2009; Abu-Hashem et al. 2011; Abu-Hashem and Aly 2012, Abu-Hashem and Badria 2015; Al-Hashem and El-Shazly 2017; Fathy et al. 2021). Also, nucleoside-triazolopyrimidine analogues exhibit different biological activities, including antiparasitic, antimicrobial, antitumor, anticonvulsant, analgesic, anti-inflammatory, and antiviral effects (Kanno et al. 1991; Lipunova et al. 2001; Nasr and Gineinah 2002; Al-Zaydi et al. 2003; Hassaneen et al. 2003; Beyer et al. 2008; Doláková et al. 2009; Li et al. 2009; Said et al. 2009; Caballero et al. 2012; Nettekoven et al. 2016; Abu-Hashem et al. 2021b). Similarly, pyridotriazolopyrimidinones exhibit critical pharmacological properties, including antioxidant, antitumor, anti-hepatitis C virus (anti-HCV), antimicrobial, anti-inflammatory, and analgesic activities, as well as the inhibition of peroxynitrite (El-Gazzar et al. 2009; Farghaly and Abdalla 2009; Farghaly et al. 2010; Riyadh et al. 2010). Thiazolyl-pyrimidine derivatives are considered to have potential anticancer activity (Abolibda et al. 2025), and antiproliferative activity (Abdelrahman et al. 2025). Quinoline is a nitrogen atom-containing fused bicyclic heterocycle known as benzo[*b*]pyridine. It is a multifunctional scaffold in medicinal chemistry, capable of forming salts with acids and undergoing electrophilic and nucleophilic substitution reactions (Yadav and Shah 2021). This characteristic enables functionalization at various positions on the ring, making it an interesting synthetic building block for drug design and synthesis. The nature and position of substituents are crucial, as they significantly influence the functionality of synthesized compounds, allowing for a broad range of applications and biological activities such as antiproliferative (Abdelbaset et al. 2018). Antimicrobial (Mubeen et al. 2018 and Abu-Hashem et al. 2023). Antihypertensive (Kumar et al. 2015). Antileishmanial (Costa et al. 2020). Antioxidant (Douadi et al. 2020). Antimalarial (Uddin et al. 2021). Anti-asthmatic (Gaurav and Singh 2014). Anticancer (Mohamed and Abuo-Rahma 2020). Antiviral (Wang et al. 2021). Anti-inflammatory (Ghate et al. 2018) and analgesic (Rajanarendar et al. 2012). Likewise, quinolines have a small molecular size and can form hydrogen bonds, making them useful in synthesising heterocycles for creating fluorescent compounds (Gu et al. 2017; Czaplinska et al. 2018). Quinoline derivatives have been utilized in diverse fields, notably in medicine (Desai et al. 2021). Also, some commercially available drugs containing the quinoline moiety **(XI–XIX)** are illustrated in Figure 2.

**Figure 3.**
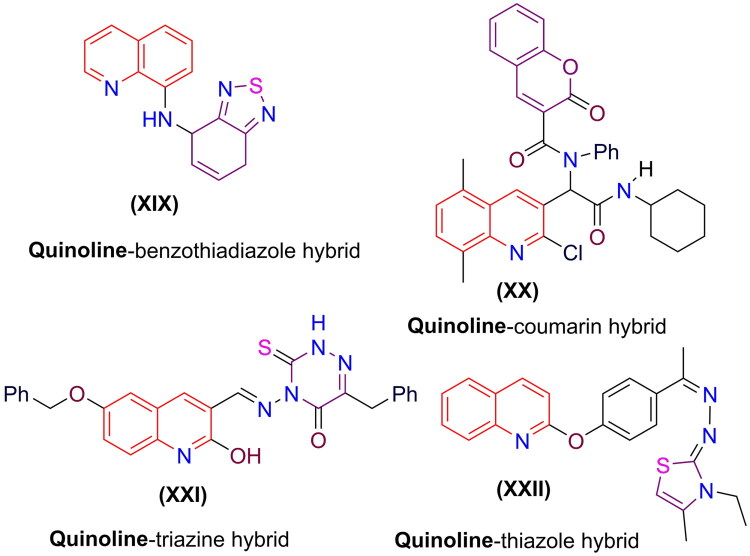
Certain quinoline hybrid structures have shown substantial biological activity.

Quinolines have garnered significant scientific interest as many researchers strive to discover more effective synthetic methods by optimizing existing reactions. The growing resistance of disease-causing organisms to currently available drugs, along with increased toxicity and side effects, has driven the development of potential drug candidates that combine two biologically active molecules into a single hybrid entity. In recent years, several quinoline hybrids have been synthesized. Research has shown that these hybrids demonstrate improved activity compared to standard drugs. Notable examples include the quinoline-benzothiadiazole hybrid **(XIX)** (Medeiros et al. 2021). The quinoline-coumarin hybrid **(XX)** (Taheri et al. 2019). The quinoline-triazine hybrid **(XXI)** (Ghanim et al. 2022). Besides the quinoline-thiazole hybrid **(XXII)**, (Eissa et al. 2021). Among others, as illustrated in Figure 3.

Molecular docking is widely used for predicting how small-molecule therapeutic compounds interact with their protein targets. This technique allows researchers to estimate the affinity and activity of these compounds. Docking plays a crucial role in rational drug design, and due to its biological and pharmacological significance, considerable efforts have been made to improve the algorithms used for docking predictions (Raval and Ganatra 2022). This approach can evaluate potential interactions between synthesized compounds and protein receptors, offering valuable insights into their binding modes and potential anticancer activity (Abouzied et al. 2022; Elmorsy et al. 2022; Alghamdi et al. 2023; Khaled et al. 2023; Sroor et al. 2024).

## Materials and methods

### Experimental section

Cooperating with other researchers, a research plan was created to synthesise new heterocyclic compounds from quinoline and pyridopyrimidine derivatives. These compounds were intended for studying their anticancer activity, and the plan was successfully executed.

### Chemistry

All melting points were determined using an Electrothermal IA 9100 series digital melting point apparatus (Shimadzu, Tokyo, Japan). Elemental analyses were performed using a Vario EL (Elementar, Langenselbold, Germany). The microanalytical data were processed at the microanalytical centre of the Faculty of Science at Cairo University and the National Research Centre. The IR spectra (KBr disc) were recorded using a Perkin-Elmer 1650 spectrometer (Waltham, MA, USA). NMR spectra were obtained using a JEOL 270 MHz and a JEOL JMS-AX 500 MHz (JEOL, Tokyo, Japan) spectrometer with Me4Si as the internal standard. Mass spectra were recorded on an EI-MS-QP 1000 EX instrument (Shimadzu, Tokyo, Japan) at 70 eV. The pharmacological evaluations of new anticancer compounds were conducted in the pharmacological unit of the Department of Pharmacognosy at the Faculty of Pharmacy, Mansoura University, Egypt. Table 1 details all starting materials, solvents, and reagents, which were obtained from Sigma-Aldrich (St. Louis, MO, USA).

**Table 1.** Clarifying the sources of materials and reagents used.

Name of materials	Source of the supplier: Sigma-Aldrich (St. Louis, MO, USA)	CAS No.	Catalogue No. (Product No.)
*N, N*-Dimethylformamide	Sigma-Aldrich	68-12-2	270547
Potassium hydroxide	Sigma-Aldrich	1310-58-3	306568
Anhydrous potassium carbonate	Sigma-Aldrich	584-08-7	590681
Anhydrous sodium acetate	Sigma-Aldrich	127-09-3	W302406
Chloroacetic acid	Sigma-Aldrich	79-11-8	402923
Glacial acetic acid	Sigma-Aldrich	64-19-7	1.00066
Formic acid	Sigma-Aldrich	64-18-6	33015
Acetic anhydride	Sigma-Aldrich	108-24-7	539996
Benzaldehyde	Sigma-Aldrich	100-52-7	B1334
4-Methoxybenzaldehyde	Sigma-Aldrich	123-11-5	8.22314
Thiophene	Sigma-Aldrich	110-02-1	T31801
2-Thiophenecarboxaldehyde	Sigma-Aldrich	98-03-3	T32409
4-(dimethyl-amino) benzaldehyde	Sigma-Aldrich	100-10-7	156477
4-chlorobenzaldehyde	Sigma-Aldrich	104-88-1	112216
Hydroxylamine hydrochloride	Sigma-Aldrich	5470-11-1	431362
Carbon disulfide	Sigma-Aldrich	75-15-0	335266
Benzoyl isothiocyanate	Sigma-Aldrich	532-55-8	261653
Benzoyl isocyanate	Sigma-Aldrich	4461-33-0	308188
1,4-Dioxane	Sigma-Aldrich	123-91-1	360481
Methanol	Sigma-Aldrich	67-56-1	34860
Ethyl alcohol	Sigma-Aldrich	64-17-5	1.11727
Dimethyl sulfoxide (DMSO)	Sigma-Aldrich	67-68-5	472301
Benzene	Sigma-Aldrich	71-43-2	270709
n-Hexane	Sigma-Aldrich	110-54-3	139386
Conc. Sulfuric acid	Sigma-Aldrich	7664-93-9	339741
Hydrochloric acid	Sigma-Aldrich	7647-01-0	258148
Fetal calf serum [Fetal Bovine Serum (FBS)]	Sigma-Aldrich	RPMI-1640 Medium	R8758

**Synthesis of 7-phenyl-5-(quinolin-2-yl)-2-thioxo-2,3-dihydropyrido[2,3-*d*] pyrimidin-4 (1*H*)-one (1)** (Abu-Hashem et al. 2023, 2024; Abu-Hashem and Al-Hussain 2024).

General Procedure: A mixture of 1-phenyl-3-(quinoline) propenone (chalcone, 2.59 g, 0.01 mol) and 6-amino-2-thioxopyrimidinone (1.43 g, 0.01 mol) in 30 mL of dimethylformamide was heated under reflux for 18–20 h. Once the reaction was complete, confirmed by thin-layer chromatography (TLC), the solution was cooled to 0 °C–5 °C, and the resulting solid was filtered out and recrystallized from DMF. The final product (**1)** was obtained as yellow crystals with an 85% yield and a melting point of >350 °C (dec.).

## Synthesis of 8-phenyl-6-(quinolin-2-yl)-5*H*-pyrido[2,3-*d*] thiazolo [3,2-a] pyrimidine-3, 5(2*H*)-dione (2)

A mixture of compound **1** (3.82 g, 0.01 mol), chloroacetic acid (0.01 mol), and anhydrous sodium acetate (0.02 mol) was gently heated with stirring in a water bath at 60-70 °C for 3-5 h, monitored by TLC. Once the reaction mixture had cooled to room temperature, it was poured into 100 mL of water. The precipitate was filtered and crystallized from dimethylformamide (DMF), producing yellow crystals. The compound **(2)** was obtained at an 84% yield, and a melting point >350 °C (decomposing); IR (*ν*, cm^−1^) KBr: 3090 (aromatic C–H stretching), 2985(CH alkyl), carbonyl groups stretching vibrations from the thiazolopyrimidine-3,5(2*H*)-dione moiety result in strong signals, observing two bands corresponding to each carbonyl group, at around 1690 and 1685 (2CO, imidic carbonyl group), 1635(C = N), 1592 (C = C); ^1^H NMR (DMSO-*d6*, ppm) *δ* 3.90 (s, 2H, CH_2_, thiazole),7.10–7.98 (m, 11H, phenyl, quinoline), 8.15 (s, 1H, pyridine); ^13^C NMR (DMSO-*d*_6_) *δ* 30.1 (1C, CH_2_), 117.5(1C, CH, pyridine), 118.9, 119.5, 120.8, 125.1, 126.9, 127.1, 127.5, 128.1, 129.3, 129.8, 137.5, 139.2, 144.4, 150.3, 154.1, 155.2, 156.1, 158.5 (20C, Ar-C),165.1, 169.4 (2C, two imidic carbonyl groups); MS (70 ev, %): *m/z* = 422 (M^+^, 100%); Anal. Calc. for C_24_H_14_N_4_O_2_S (422.46): C, 68.23 (68.30); H, 3.34 (3.38); N, 13.26 (13.21).

## Synthesis of 2-substituted-8-phenyl-6-(quinolin-2-yl)-5*H*-pyrido[2,3-*d*] thiazolo [3,2-*a*] pyrimidine-3,5(2*H*)-dione (3a-e)

**General Procedure: Method (A):** A mixture was prepared by combining compound **1** (3.82 g, 0.01 mol), chloroacetic acid (0.01 mol), the appropriate aromatic aldehyde (0.01 mol), and 0.02 mol of anhydrous sodium acetate. This mixture was stirred and refluxed in 40 mL of glacial acetic acid and 20 mL of acetic anhydride for 20 h, as monitored by TLC. After the reaction, the mixture was allowed to cool and poured onto 100 mL of cold water. The resulting precipitate was filtered and crystallized from a suitable solvent to produce compounds **(3a–e)**.

**Method (B):** A mixture of **2** (4.22 g, 0.01 mol), aromatic aldehyde (0.01 mol), and 0.02 mol of anhydrous sodium acetate was stirred under reflux in 40 mL of glacial acetic acid and 20 mL of acetic anhydride for 6–8 h, while monitoring the reaction with TLC. Once the reaction was complete, the mixture was allowed to cool to room temperature and poured onto 100 mL of cold water. The resulting precipitate was filtered and crystallized from an appropriate solvent, yielding products **(3a–e)**.

## Synthesis of 2-benzylidene-8-phenyl-6-(quinolin-2-yl)-5*H*-pyrido[2,3-*d*] thiazolo[3,2-*a*] pyrimidine-3,5(2*H*)-dione (3a)

The yellowish crystals were obtained by reacting either compound **1** (3.82 g, 0.01 mol) or compound **2** (4.22 g, 0.01 mol) with benzaldehyde (1.06 g, 0.01 mol). Compound **(3a)** was crystallized from dioxane with a high yield of 81%, and a melting point exceeding 350 °C (dec.); IR (*ν*, cm^−1^) KBr: 3085 (CH aryl), 2970 (CH alkyl),1695, 1683 (2CO, imidic carbonyl), 1637 (C = N), 1588 (C = C); ^1^H NMR (DMSO-*d6*, ppm) *δ* 7.10–8.10 (m, 16H, two phenyls, quinoline), 8.20 (s, 1H, methine proton), 8.30 (s, 1H, pyridine); ^13^C NMR (DMSO-*d*_6_) *δ* 110.5 (1C, thiazole), 110.8 (1C, CH, pyridine),143.0 (1C, CH, methene proton), 112.1, 112.2, 112.4, 113.1, 113.2, 113.5, 113.8, 113.9, 119.4, 125.7, 128.7, 130.5, 130.7, 131.4, 135.7, 148.6, 152.9, 158.1, 158.2, 158.4, 158.5, 158.7 (26 C, Ar–C),163.0, 167.2 (2C, two imidic carbonyl groups); MS (70 ev, %): *m/z* = 510 (M^+^, 100%); Anal. Calc. for C_31_H_18_N_4_O_2_S (510.57): C, 72.93 (72.88); H, 3.55 (3.60); N, 10.97 (10.92).

## Synthesis of 2-(4-chlorobenzylidene)-8-phenyl-6-(quinolin-2-yl)-5*H*-pyrido[2,3-*d*] thiazolo [3, 2-a] pyrimidine-3,5(2*H*)-dione (3b)

The yellow crystals were obtained by reacting either compound **1** (3.82 g, 0.01 mol) or compound **2** (4.22 g, 0.01 mol) with 4-chlorobenzaldehyde (1.40 g, 0.01 mol). The compound was crystallized from methanol with a high yield of 85%, and a melting point > 350 °C (dec.); IR (*ν*, cm^−1^) KBr: 3090 (CH aryl), 2980 (CH alkyl),1697, 1688 (2CO, imidic carbonyl), 1638(C = N), 1590 (C = C); ^1^H NMR (DMSO-*d6*, ppm) *δ* 7.05 − 7.96 (m, 15H, two phenyls, quinoline), 8.05 (s, 1H, methine proton); 8.12 (s, 1H, pyridine); ^13^C NMR (DMSO-*d*_6_) *δ* 116.1 (1 C, thiazole), 117.8 (1C, CH, pyridine),142.7 (1C, CH, methene proton), 118.5, 119.1, 121.2, 125.6, 127.2, 127.3, 127.5, 128.2, 128.5, 129.1, 129.5, 130.1, 133.2, 133.7, 137.4, 139.1, 144.5, 150.3, 154.1, 155.2, 156.6, 158.4 (26 C, Ar–C),169.1, 171.4 (2C, two imidic carbonyl groups); MS (70 ev, %): *m/z* = 545 (M^+^, 100%); Anal. Calc. for C_31_H_17_ClN_4_O_2_S (545.01): C, 68.32(68.38); H, 3.14 (3.20); N, 10.28 (10.22).

## Synthesis of 2-(4-methoxybenzylidene)-8-phenyl-6-(quinolin-2-yl)-5*H*-pyrido[2,3-*d*] thiazolo [3,2-*a*] pyrimidine-3,5(2*H*)-dione (3c)

The yellowish crystals were obtained by reacting either compound **1** (3.82 g, 10 mmol) or compound **2** (4.22 g, 10 mmol) with 4-methoxybenzaldehyde (1.36 g, 10 mmol). The compound was crystallized from ethanol, with a good yield of 84%, and a melting point > 350 °C (dec.); IR (*ν*, cm^−1^) KBr: 3092 (CH aryl), 2987(CH alkyl),1692, 1683 (2CO, amide), 1634(C = N), 1591 (C = C); ^1^H NMR (DMSO-*d6*, ppm) *δ* 3.75 (s, 3H, OCH_3_), 7.02–7.93 (m, 15H, two phenyls, quinoline), 8.07 (s, 1H, methine proton); 8.16 (s, 1H, pyridine); ^13^C NMR (DMSO-*d*_6_) *δ* 53.5 (1 C, OCH_3_), 116.4 (*1C, thiazole*), 117.5 (*1C, CH*, pyridine),142.9 (*1C, CH, methene* proton), 118.7, 119.4, 120.5, 121.2, 125.2, 127.1, 127.4, 127.6, 127.8, 128.1, 129.4, 129.8, 130.5, 137.5, 139.2, 144.8, 150.5, 154.2, 155.3, 156.1, 158.2, 159.7 (26 C, Ar–C),168.9, 171.1 (2C, two imidic carbonyl groups); MS (70 ev, %): *m/z* = 540 (M^+^, 100%); Anal. Calc. for C_32_H_20_N_4_O_3_S (540.60): C, 71.10(71.15); H, 3.73 (3.78); N, 10.36 (10.30).

## Synthesis of 8-phenyl-6-(quinolin-2-yl)-2-(thiophen-2-ylmethylene)-5*H*-pyrido[2,3-*d*] thiazolo [3, 2-a] pyrimidine-3,5(2*H*)-dione (3d)

The yellow crystals were obtained by reacting either compound **1** (3.82 g, 0.01 mol) or compound **2** (4.22 g, 0.01 mol) with thiophene-2-carbaldehyde (1.12 g, 0.01 mol). The compound was crystallized from DMF, with a good yield of 80%, and a melting point > 350 °C (dec.); IR (*ν*, cm^−1^) KBr: 3086 (CH aryl), 2980(CH alkyl),1690, 1682 (2CO, imidic carbonyl), 1632(C = N), 1585 (C = C); ^1^H NMR (DMSO-*d6*, ppm) *δ* 5.20 (d, 1H, *J =* 6.4 Hz, thiophene), 5.40 (t, 1H, *J =* 6.4 Hz, thiophene), 5.60 (d, 1H, *J =* 6.4 Hz, thiophene), 7.15 − 7.90 (m, 11H, phenyl, quinoline), 8.09 (s, 1H, methine proton); 8.18 (s, 1H, pyridine); ^13^C NMR (DMSO-*d*_6_) *δ* 116.7 (1C, thiazole), 117.2 (1C, CH, pyridine),143.1 (1C, CH, methene proton), 118.2, 119.1, 121.4, 125.3, 126.9, 127.2, 127.7, 128.2, 128.4, 129.2, 129.4, 129.7, 130.6, 136.9, 137.7, 139.1, 144.5, 150.6, 154.1, 154.8, 156.3, 158.5 (24C, Ar–C),168.5, 171.7 (2C, two, imidic carbonyl groups); MS (70 ev, %): *m/z* = 516 (M^+^, 100%); Anal. Calc. for C_29_H_16_N_4_O_2_S_2_ (516.59): C, 67.43(67.50); H, 3.12 (3.08); N, 10.85 (10.80).

## Synthesis of 2-(4-(dimethylamino) benzylidene)-8-phenyl-6-(quinolin-2-yl)-5*H*-pyrido[2,3-*d*] thiazolo [3, 2-a] pyrimidine-3,5(2*H*)-dione (3e)

The yellowish crystals were obtained by reacting either compound **1** (3.82 g, 0.01 mol) or compound **2** (4.22 g, 10 mmol) with 4-(dimethylamino) benzaldehyde (1.49 g, 0.01 mol). The compound was crystallized from dioxane, with a good yield of 79%, and a melting point > 350 °C (dec.); IR (*ν*, cm^−1^) KBr: 3085 (CH aryl), 2975 (CH alkyl),1694, 1680 (2CO, imidic carbonyl groups), 1630(C = N), 1582 (C = C); ^1^H NMR (DMSO-*d6*, ppm) *δ* 2.90 (s, 6H, 2CH_3_), 7.07 − 7.98 (m, 15H, two phenyls, quinoline), 8.07 (s, 1H, methine proton); 8.18 (s, 1H, pyridine); ^13^C NMR (DMSO-*d*_6_) *δ* 37.5 (2C, 2CH_3_), 116.2 (1C, thiazole), 117.9 (1C, CH, pyridine), 142.5 (1C, CH, methene proton), 118.3, 119.4, 120.2, 121.1, 124.5, 125.7, 127.1, 127.4, 127.8, 128.1, 129.3, 129.6, 129.8, 137.5, 139.2, 144.4, 150.1, 150.5, 154.2, 155.3, 156.1, 158.2 (26C, Ar–C),167.5, 171.8 (2 C, two, imidic carbonyl groups); MS (70 ev, %): *m/z* = 553 (M^+^, 90%); Anal. Calc. for C_33_H_23_N_5_O_2_S (553.64): C, 71.59 (71.52); H, 4.19 (4.25); N, 12.65 (12.58).

## Synthesis of 3-(substituted)-7-phenyl-9-(quinolin-2-yl)-2,3-dihydro-10*H*-isoxazolo [5′,4′:4,5] thiazolo [3,2-*a*] pyrido[2,3-*d*] pyrimidin-10-one (5a-e)

A mixture of compounds **3a–c** (0.01 mol), hydroxylamine hydrochloride (10 mmol), and anhydrous sodium acetate (0.01 mol) was stirred and refluxed in 30 mL of glacial acetic acid for 8–12 h, with the reaction monitored by TLC. After the reaction, the mixture was allowed to cool to room temperature and poured onto 100 mL of cold water. The resulting precipitate was filtered, dried and recrystallized from an appropriate solvent to yield products **5a–c**.

## Synthesis of 3,7-diphenyl-9-(quinolin-2-yl)-2,3-dihydro-10*H*-isoxazolo [5′,4′:4,5] thiazolo[3,2-*a*] pyrido[2,3-*d*] pyrimidin-10-one (5a)

The yellow crystals were obtained by reacting compound **3a** (5.10 g, 0.01 mol) with hydroxylamine hydrochloride (0.69 g, 0.01 mol). The compound was crystallized from DMF, with a good yield (77%), and a melting point >350 °C (dec.); IR (*ν*, cm^−1^) KBr: 3310 (NH), 3082 (CH aryl), 2973 (CH alkyl),1684 (CO, imidic carbonyl group), 1628(C = N), 1575 (C = C); ^1^H NMR (DMSO-*d6*, ppm) *δ* 4.20 (d, 1H, *J =* 6.4 Hz, iso-oxazole proton), 7.10–8.00 (m, 16H, two phenyls, quinoline), 8.10 (s, 1H, pyridine), 9.90 (s, NH, D_2_O exchangeable); ^13^C NMR (DMSO-*d*_6_) *δ* 63.8, 64.4 (2C, isoxazole), 112.6 (1C, CH, pyridine), 115.1, 115.5, 115.6, 115.9, 116.1, 116.4, 116.7, 116.8, 116.9,117.1, 117.2, 119.2, 122.4,125.7, 129.0, 131.2, 131.3, 135.8, 143.2, 143.3, 147.7, 158.2, 159.6 (27 C, Ar–C),165.7 (1C, one imidic carbonyl group); MS (70 ev, %): *m/z* = 525 (M^+^, 100%); Anal. Calc. for C_31_H_19_N_5_O_2_S (525.59): C, 70.84 (70.90); H, 3.64 (3.58); N, 13.33 (13.40).

## Synthesis of 3-(4-chlorophenyl)-7-phenyl-9-(quinolin-2-yl)-2, 3-dihydro-10*H*-isoxazolo [5′, 4′: 4, 5] thiazolo [3, 2-a] pyrido[2,3-*d*] pyrimidin-10-one (5b)

The yellowish crystals were obtained by reacting compound **3b** (5.45 g, 0.01 mol) with hydroxylamine hydrochloride (0.69 g, 0.01 mol). The compound was crystallized from tetrahydrofuran (THF), with a good yield (75%), and a melting point >350 °C (dec.); IR (*ν*, cm^−1^) KBr: 3315 (NH), 3080 (CH aryl), 2970 (CH alkyl),1680 (CO, imidic carbonyl group), 1629 (C = N), 1578 (C = C); ^1^H NMR (DMSO-*d6*, ppm) *δ* 5.60 (d, 1H, *J =* 6.4 Hz, iso-oxazole proton), 7.01–7.95 (m, 15H, two phenyls, quinoline), 8.16 (s, 1H, pyridine),10.20 (s, NH, D_2_O exchangeable); ^13^C NMR (DMSO-*d*_6_) *δ* 65.8, 71.7 (2C, isoxazole), 117.9 (1C, CH, pyridine), 118.7, 119.4, 121.5, 125.1, 126.3, 127.1, 127.2, 127.8,128.1, 128.8, 129.3, 129.9, 132.5, 137.4, 139.1, 141.5, 144.6, 150.2, 154.3, 155.1, 156.4, 158.5, 161.2 (27C, Ar–C),166.5 (1C, one carbonyl group); MS (70 ev, %): *m/z* = 560 (M^+^, 100%); Anal. Calc. for C_31_H_18_ClN_5_O_2_S (560.03): C, 66.49 (66.42); H, 3.24 (3.30); N, 12.51 (12.57).

## Synthesis of 3-(4-methoxyphenyl)-7-phenyl-9-(quinolin-2-yl)-2,3-dihydro-10*H*-isoxazolo [5′,4′:4,5] thiazolo [3,2-*a*] pyrido [2,3-*d*] pyrimidin-10-one (5c)

The brownish crystals were obtained by reacting compound **3c** (5.40 g, 0.01 mol) with hydroxylamine hydrochloride (0.69 g, 0.01 mol). The compound was crystallized from dioxane, with a good yield (76%), and a melting point >350 °C (dec.); IR (*ν*, cm^−1^) KBr: 3320 (NH), 3078 (CH aryl), 2972 (CH alkyl),1681 (CO, imidic carbonyl group), 1628 (C = N), 1575 (C = C); ^1^H NMR (DMSO-*d6*, ppm) *δ* 3.80 (s, 3H, OCH_3_), 5.65 (d, 1H, *J =* 6.3 Hz, iso-oxazole proton), 7.04–7.98 (m, 15H, two phenyls, quinoline), 8.20 (s, 1H, pyridine),10.25 (s, NH, D_2_O exchangeable); ^13^C NMR (DMSO-*d*_6_) *δ* 54.1 (1C, OCH_3_), 66.2, 70.8 (2 C, isoxazole), 117.5 (1C, CH, pyridine), 118.3, 119.4, 120.7, 121.5, 125.1, 126.1, 127.2, 127.4, 127.9,128.2, 129.1, 129.8, 134.8, 137.5, 139.3, 144.4, 150.5, 154.1, 155.3, 156.1, 158.5, 158.8, 161.4 (27C, Ar–C),166.8 (1C, one imidic carbonyl group); MS (70 ev, %): *m/z* = 555 (M^+^, 100%); Anal. Calc. for C_32_H_21_N_5_O_3_S (555.61): C, 69.18 (69.25); H, 3.81 (3.75); N, 12.61(12.66).

## Synthesis of 7-phenyl-9-(quinolin-2-yl)-3-(thiophen-2-yl)-2,3-dihydro-10*H*-isoxazolo [5′,4′: 4, 5] thiazolo[3,2-*a*] pyrido [2,3-*d*] pyrimidin-10-one (5d)

The yellowish crystals were obtained by reacting compound **3d** (5.16 g, 0.01 mol) with hydroxylamine hydrochloride (0.69 g, 0.01 mol). The compound was crystallized from n-hexane, with a good yield (73%), and a melting point >350 °C (dec.); IR (*ν*, cm^−1^) KBr: 3325 (NH), 3084 (CH aryl), 2976 (CH alkyl),1684 (CO, imidic carbonyl group), 1631 (C = N), 1579 (C = C); ^1^H NMR (DMSO-*d6*, ppm) *δ* 5.30 (d, 1H, *J =* 6.5 Hz, thiophene), 5.50 (t, 1H, *J =* 6.5 Hz, thiophene), 5.66 (d, 1H, *J =* 6.4 Hz, thiophene), 5.75 (d, 1H, *J =* 6.3 Hz, iso-oxazole proton), 7.11–7.90 (m, 11H, phenyl, quinoline), 8.16 (s, 1H, pyridine), 10.30 (s, NH, D_2_O exchangeable); ^13^C NMR (DMSO-*d*_6_) *δ* 66.5, 71.3 (2C, isoxazole), 117.1 (1C, CH, pyridine), 118.4, 119.3, 121.1, 124.8, 125.6, 126.8, 127.1, 127.2, 127.5, 127.8,128.1, 129.4, 129.7, 136.9, 138.8, 139.5, 144.2, 150.8, 154.2, 155.1, 156.3, 158.7, 161.5 (25C, Ar–C),166.3 (1C, one imidic carbonyl group); MS (70 ev, %): *m/z* = 531 (M^+^, 100%); Anal. Calc. for C_29_H_17_N_5_O_2_S_2_ (531.61): C, 65.52 (65.60); H, 3.22 (3.28); N, 13.17(13.10).

## Synthesis of 3-(4-(dimethyl-amino) phenyl)-7-phenyl-9-(quinolin-2-yl)-2,3-dihydro-10*H*-isoxazolo [5′, 4′:4, 5] thiazolo[3,2-a] pyrido[2,3-*d*] pyrimidin-10-one (5e)

The yellow crystals were obtained by reacting compound **3e** (5.53 g, 0.01 mol) with hydroxylamine hydrochloride (0.69 g, 0.01 mol). The compound was crystallized from methanol, with a good yield (71%), and a melting point >350 °C (dec.); IR (*ν*, cm^−1^) KBr: 3327 (NH), 3089 (CH aryl), 2985 (CH alkyl),1686 (CO, imidic carbonyl group), 1632 (C = N), 1588 (C = C); ^1^H NMR (DMSO-*d6*, ppm) *δ* 3.10 (s, 6H, 2CH_3_), 5.80 (d, 1H, *J =* 6.3 Hz, iso-oxazole proton), 7.07–7.99 (m, 15H, two phenyls, quinoline), 8.25 (s, 1H, pyridine),10.35 (s, NH, D_2_O exchangeable); ^13^C NMR (DMSO-*d*_6_) *δ* 33.5 (2C, 2CH_3_), 68.1, 71.4 (2C, isoxazole), 117.6 (1C, CH, pyridine), 118.3, 119.5, 120.2, 121.3, 125.4, 126.5, 127.1, 127.2, 127.8,128.4, 129.3, 129.7, 133.1, 137.2, 139.1, 144.5, 149.5, 150.2, 154.4, 155.1, 156.5, 158.5, 161.3 (27C, Ar–C),167.1 (1C, one imidic carbonyl group); MS (70 ev, %): *m/z* = 568 (M^+^, 100%); Anal. Calc. for C_33_H_24_N_6_O_2_S (568.65): C, 69.70 (69.75); H, 4.25 (4.20); N, 14.78 (14.71).

## Synthesis of 3-amino-5-oxo-8-phenyl-6-(quinolin-2-yl)-5*H*-pyrido[2,3-*d*] thiazolo [3, 2-a] pyrimidine-2-carbonitrile (6)

A mixture containing compound **1** (3.82 g, 0.01 mol), bromomalononitrile (1.45 g, 0.01 mol), and anhydrous potassium carbonate (2.76 g, 20 mmol) was stirred under reflux in 40 mL of ethanol for 11 to 15 h, monitoring the reaction by TLC. After the reaction, the mixture was allowed to cool to room temperature and poured onto 100 mL of cold water. The precipitate was filtered, dried, and crystallized from DMF, resulting in the formation of yellowish crystals of product **(6)** with an 82% yield. The product’s melting point was more than >350 °C (dec.); IR (*ν*, cm^−1^) KBr: 3426-3421 (NH_2_), 3091 (CH aryl), 2987 (CH alkyl), 2215 (CN), 1692 (CO, imidic carbonyl group), 1631(C = N), 1584 (C = C); ^1^H NMR (DMSO-*d6*, ppm) *δ* 7.10–8.00 (m, 11H, phenyl, quinoline), 8.10 (s, 1H, pyridine), *δ* 9.90 a broad singlet signal (br.s, NH_2,_ D_2_O exchangeable); ^13^C NMR (DMSO-*d*_6_) *δ* 56.3 (1C, thiazole), 105.7 (1C, CN),106.7 (1C, CH, pyridine), 112.1, 117.1, 117.2, 118.2, 119.2, 122.4, 124.7, 125.7, 128.8, 130.5, 130.6, 135.3, 135.8, 141.4, 143.3, 147.7, 149.0, 157.9, 158.2 (21C, Ar-C),167.7 (1C, one imidic carbonyl group); MS (70 ev, %): *m/z* = 446 (M^+^, 100%); Anal. Calc. for C_25_H_14_N_6_OS (446.49): C, 67.25(67.32); H, 3.16 (3.22); N, 18.82 (18.75).

## Synthesis of substituted-8-phenyl-10-(quinolin-2-yl)-pyrimido [4′, 5′: 4,5] thiazolo [2, 3-b] pyrido [3″, 2″- e] pyrimidine-4, 11(3*H*)-dione (7a, b)

**General Procedure: Method (A):** Compound **6** (4.46 g, 10 mmol), formic acid (10 mL) or acetic acid (10 mL) and a catalytic amount of concentrated hydrochloric acid were heated under reflux for 15–18 h. The reaction mixture was allowed to cool to room temperature and poured onto cold water (100 mL). The formed solid was collected by filtration, washed with ethanol (20 mL), dried and crystallized from a suitable solvent.

**Method (B):** A mixture of compound **6** (4.46 g, 0.01 mol) and formic acid (30 mL) or acetic acid (30 mL) was heated under reflux for 20 h. The reaction mixture was allowed to cool to room temperature and poured onto cold water (100 mL). The formed solid was collected by filtration, washed with ethanol (20 mL), dried and crystallized from a suitable solvent.

## Synthesis of 8-phenyl-10-(quinolin-2-yl)-pyrimido[4′,5′:4,5] thiazolo [2,3-b] pyrido[3″,2″-e] pyrimidine-4,11(3*H*)-dione (7a)

The yellow crystals were obtained by reacting compound **6** (4.46 g, 0.01 mol) and formic acid (10 mL). The compound was crystallized from dimethylformamide, with an (84%) yield, and a melting point >350 °C (dec.); IR (*ν*, cm^−1^) KBr: 3250 (NH), 3088 (CH aryl), 2970 (CH alkyl), 1685, 1680 (2CO, two imidic carbonyl group), 1627(C = N), 1580 (C = C); ^1^H NMR (DMSO-*d6*, ppm) *δ* 7.0–8.0 (m, 11H, phenyl, quinoline), 8.10 (s, 1H, pyridine), 8.40 (s, 1H, pyrimidine), 9.0 (s, NH_,_ D_2_O exchangeable); ^13^C NMR (DMSO-*d*_6_) *δ* 112.0 (1C, CH, pyridine), 147.7 (1C, CH, pyrimidine), 112.1, 112.2, 114.4, 117.1, 1117.2, 118.2, 119.1, 119.2, 122.4, 125.0, 125.5, 126.9, 128.9, 129.0, 131.2, 131.3, 135.8, 143.2, 143.3, 158.2, (22C, Ar–C), 164.4, 166.8 (2 C, two imidic carbonyl groups); MS (70 ev, %): *m/z* = 474 (M^+^, 100%); Anal. Calc. for C_26_H_14_N_6_O_2_S (474.50): C, 65.81(65.75); H, 2.97 (2.92); N, 17.71 (17.85).

## Synthesis of 2-methyl-8-phenyl-10-(quinolin-2-yl)-pyrimido[4′,5′:4,5] thiazolo [2,3-*b*] pyrido [3″, 2″ -e] pyrimidine-4,11(3*H*)-dione (7b)

The brown crystals were obtained by reacting compound **6** (4.46 g, 0.01 mol) and acetic acid (10 mL). The compound was crystallized from ethanol, with an (81%) yield; and a melting point greater than 350 °C (dec.); IR (*ν*, cm^−1^) KBr: 3260 (NH), 3095 (CH aryl), 2980 (CH alkyl), 1690, 1684 (2CO, two imidic carbonyl group), 1629(C = N), 1583 (C = C); ^1^H NMR (DMSO-*d6*, ppm) *δ* 2.20 (s, 3H, CH_3_), 7.05–7.88 (m, 11H, phenyl, quinoline), 8.25 (s, 1H, pyridine), 11.70 (s, NH_,_ D_2_O exchangeable); ^13^C NMR (DMSO-*d*_6_) *δ* 24.7 (1C, CH_3_), 115.8 (1C, CH, pyridine), 116.2, 117.5, 118.9, 120.8, 124.7, 126.8, 127.1, 127.5, 128.2, 129.4, 130.2, 137.8, 138.9, 144.5, 151.1, 153.5, 154.3, 154.8, 155.9, 156.8, 158.6, (23C, Ar–C), 161.1, 167.3 (2 C, two imidic carbonyl groups); MS (70 ev, %): *m/z* = 488 (M^+^, 100%); Anal. Calc. for C_27_H_16_N_6_O_2_S (488.52): C, 66.38 (66.31); H, 3.30 (3.37); N, 17.20 (17.15).

## Synthesis of 4-amino-8-phenyl-10-(quinolin-2-yl)-pyrimido[4′,5′:4,5] thiazolo [2,3-b] pyrido [3″, 2″-e] pyrimidine-11-one (8)

**Method (A):** A mixture of compound **6** (4.46 g, 0.01 mol), formamide (10 mL), and formic acid (2 mL) was stirred under reflux in dimethylformamide (50 mL) for 5–8 h. The reaction mixture was allowed to cool to room temperature and poured onto water (100 mL), neutralized with an ammonia solution. The precipitate was filtered, washed with water and ethanol, dried, and crystallized from dioxane.

**Method (B):** To a warmed ethanolic sodium ethoxide solution (prepared by dissolving 0.23 g (10 mmol) of sodium metal in 50 mL of absolute ethanol), 4.46 g (0.01 mol) of compound (6) and formic acid (2 mL) were added. The mixture was stirred under reflux for 5–7 h. The reaction mixture was allowed to cool to room temperature, poured onto cold water (100 mL), and neutralized with acetic acid. The deposited so-precipitate was filtered, dried and crystallized from dioxane. Yellow crystals, yield (90%), and a melting point greater than 350 °C (dec.); IR (*ν*, cm^−1^) KBr: 3401–3396 (NH_2_), 3094 (CH aryl), 2950 (CH alkyl),1677 (CO, imidic carbonyl group), 1625(C = N), 1575 (C = C); ^1^H NMR (DMSO-*d6*, ppm) *δ* 6.30 a broad singlet signal (br.s, NH_2_, D_2_O exchangeable), 7.0–7.90 (m, 11H, phenyl, quinoline), 8.10 (s, 1H, pyridine), 8.20 (s, 1H, pyrimidine); ^13^C NMR (DMSO-*d*_6_) *δ* 114.9 (1C, CH, pyridine), 159.1 (1C, CH, pyrimidine),115.5, 116.9, 118.2, 120.4, 124.7, 126.8, 127.1, 127.5, 128.1, 129.1, 130.2, 137.1, 139.4, 144.3, 150.5, 154.2, 155.1, 156.1, 157.1, 157.8, 158.6 (23C, Ar-C), 161.4 (1C, imidic carbonyl group); MS (70 ev, %): *m/z* = 473 (M^+^, 100%); Anal. Calc. for C_26_H_15_N_7_OS (473.51): C, 65.95 (65.90); H, 3.19 (3.27); N, 20.71 (20.63).

## Synthesis of 4-imino-8-phenyl-10-(quinolin-2-yl)-2-thioxo-1,4-dihydro-2*H*,11*H*-pyrido [2″,3″:4′,5′] pyrimido[2′,1′:2, 3] thiazolo[4,5-d] [1,3] thiazin-11-one (9)

A mixture of compound **6** (2.23 g, 0.005 mol) and carbon disulfide (excess, 10 mL) was heated under reflux on a water bath (80 °C) in 40 mL of pyridine for 10-12 h (TLC control). The reaction mixture was allowed to cool to 0 °C for 13–15 h; the precipitate was filtered, washed with ethanol (40 mL), dried and crystallized from ethanol. Yellowish crystals, yield (75%), and a melting point > 350 °C (dec.); IR (*ν*, cm^−1^) KBr: 3290–3270 (2NH), 3084 (CH aryl), 2945 (CH alkyl),1673 (CO, imidic carbonyl group), 1628(C = N), 1583 (C = C), 1352 (C = S); ^1^H NMR (DMSO-*d6*, ppm) *δ* 7.10–7.90 (m, 11H, phenyl, quinoline), 8.10 (s, 1H, pyridine), 9.10 (s, NH, D_2_O exchangeable), 9.90 a broad singlet signal (s, NH_,_ D_2_O exchangeable); ^13^C NMR (DMSO-*d*_6_) *δ* 115.5 (1 C, CH, pyridine),116.2, 117.1, 118.8, 120.9, 124.6, 126.9, 127.2, 127.8, 128.2, 129.4, 130.1, 137.5, 139.1, 143.9, 144.5, 150.2, 154.1, 155.4, 156.5, 158.7, 161.7 (23C, Ar–C), 164.9 (1C, imidic carbonyl group),185.8 (1C, C = S, group); MS (70 ev, %): *m/z* = 522 (M^+^, 100%); Anal. Calc. for C_26_H_14_N_6_OS_3_ (522.62): C, 59.75 (59.82); H, 2.70 (2.77); N, 16.08 (16.15).

## Synthesis of *N*-((2-cyano-5-oxo-8-phenyl-6-(quinolin-2-yl)-5*H*-pyrido[2,3-*d*] thiazolo[3,2-a] pyrimidin-3-yl) carbamothioyl or carbamoyl) benzamide (10a, b)

To a solution of compound **6** (4.46 g, 0.01 mole) in 40 mL of dry benzene, 0.015 moles of (1.40 mL of benzoyl isothiocyanate or 1.35 mL of benzoyl isocyanate) was added dropwise. The mixture was refluxed for 6–9 h under control (TLC). After cooling, the precipitated crystals were filtered, washed with ether, and recrystallized from a suitable solvent, such as toluene or benzene.

## Synthesis of *N*-((2-cyano-5-oxo-8-phenyl-6-(quinolin-2-yl)-5*H*-pyrido[2,3-d] thiazolo [3,2-a] pyrimidin-3-yl) carbamothioyl) benzamide (10a)

The yellowish crystals were obtained by reacting compound **6** (4.46 g, 0.01 mol) and 0.015 moles of (1.40 mL) benzoyl isothiocyanate. The compound was crystallized from toluene, with an (80%) yield, and a melting point > 350 °C (dec.); IR (*ν*, cm^−1^) KBr: 3200-3310 (2NH), 3091 (CH aryl), 2979 (CH alkyl), 2230(CN), 1695, 1684 (2CO, two imidic carbonyl group), 1631(C = N), 1583 (C = C), 1200 (C = S); ^1^H NMR (DMSO-*d6*, ppm) *δ* 6.98–7.99 (m, 16H, two phenyl, quinoline), 8.28 (s, 1H, pyridine), 11.03 (s, NH_,_ D_2_O exchangeable), 12.20 (s, NH_,_ D_2_O exchangeable); ^13^C NMR (DMSO-*d*_6_) *δ* 74.7 (1 C, thiazole), 103.9 (1C, CN), 104.1 (1C, CH, pyridine), 104.2, 115.7, 115.8, 117.3, 117.4, 127.5, 127.6, 129.4, 129.5, 129.6, 133.2, 133.4, 133.5, 135.1, 137.2, 138.4, 138.5, 138.6, 149.6, 151.2, 154.8, 154.9, 160.8 (27C, Ar–C), 162.4, 171.8 (2C, two imidic carbonyl groups), 171.9 (1C, C = S, group); MS (70 ev, %): *m/z* = 609 (M^+^, 99%); Anal. Calc. for C_33_H_19_N_7_O_2_S_2_ (609.68): C, 65.01(65.10); H, 3.14 (3.07); N, 16.08 (16.15).

## Synthesis of *N*-((2-cyano-5-oxo-8-phenyl-6-(quinolin-2-yl)-5H-pyrido[2,3-*d*] thiazolo[3,2-*a*] pyrimidin-3-yl) carbamoyl) benzamide (10b)

The brownish crystals were obtained by reacting compound **6** (4.46 g, 0.01 mol) and 0.015 moles of (1.35 mL) benzoyl isocyanate. The compound was crystallized from benzene, with an (72%) yield, and a melting point > 350 °C (dec.); IR (*ν*, cm^−1^) KBr: 3220-3320 (2NH), 3095 (CH aryl), 2988 (CH alkyl), 2235(CN), 1697, 1688, 1680 (3CO, three imidic carbonyl groups), 1633(C = N), 1585 (C = C); ^1^H NMR (DMSO-*d6*, ppm) *δ* 7.05–8.05 (m, 16H, two phenyl, quinoline), 8.25 (s, 1H, pyridine), 9.10 (s, NH_,_ D_2_O exchangeable), 11.70 (s, NH_,_ D_2_O exchangeable); ^13^C NMR (DMSO-*d*_6_) *δ* 75.5 (1C, thiazole), 113.1 (1C, CN), 116.3 (1C, CH, pyridine), 118.4, 119.5, 121.1, 125.2, 126.9, 127.1, 127.4, 127.9, 128.1, 128.7, 129.3, 130.1, 132.2, 133.5, 137.2, 139.4, 144.1, 146.4, 150.2, 154.1, 155.2, 156.3, 158.5 (27C, Ar–C), 160.4, 166.1, 167.5 (3C, three imidic carbonyl groups); MS (70 ev, %): *m/z* = 593 (M^+^, 95%); Anal. Calc. for C_33_H_19_N_7_O_3_S (593.62): C, 66.77 (66.70); H, 3.23 (3.30); N, 16.52 (16.45).

## Synthesis of 4-amino-3-benzoyl-2-substituted-8-phenyl-10-(quinolin-2-yl)-pyrimido [4′,5′:4,5] thiazolo [2,3-b] pyrido[3″,2″-e] pyrimidine-11-one (11a, b)

**General method A:** A mixture of compound **6** (4.46 g, 0.01 mole) and benzoyl isothiocyanate (1.35 mL, 0.01 mol) or benzoyl isocyanate (1.30 mL, 0.01 mol) in pyridine (40 mL) was refluxed for 11–14 h under control (TLC). The reaction mixture was left to cool and acidified with dilute hydrochloric acid, and the formed product was. The precipitate was filtered, dried and recrystallized from a suitable solvent to give **11a** and **11b**, respectively.

**Method B:** A mixture of 0.01 mole of compounds (**10a** or **10b)** and (50 mL of a 1.5% aqueous potassium hydroxide solution) or (50 mL of a 1% ethanol solution of potassium hydroxide) was refluxed for 9–12 h under control (TLC). After cooling, the solution was neutralized with an 18% hydrochloric acid solution, and the precipitated crystals were filtered, washed with water, and recrystallized from the proper solvent to give **11a** and **11b**, respectively.

## Synthesis of 4-amino-3-benzoyl-2-thioxo-8-phenyl-10-(quinolin-2-yl)-pyrimido [4′,5′:4,5] thiazolo [2,3-*b*] pyrido[3″,2″-*e*] pyrimidine-11-one (11a)

The pale-yellow crystals were obtained by reacting compound **6** (4.46 g, 0.01 mol) and benzoyl isothiocyanate (1.35 mL, 0.01 mol). The compound was crystallized from dioxane, with an (78%) yield, and a melting point > 350 °C (dec.); IR (*ν*, cm^−1^) KBr: 3411–3406 (NH_2_), 3097 (CH aryl), 2988(CH alkyl),1698, 1690 (2CO, two imidic carbonyl groups), 1635(C = N), 1591 (C = C), 1210 (C = S); ^1^H NMR (DMSO-*d6*, ppm) *δ* 6.78 a broad singlet signal (br.s, NH_2,_ D_2_O exchangeable), 7.10–7.96 (m, 16H, two phenyl, quinoline), 8.16 (s, 1H, pyridine); ^13^C NMR (DMSO-*d*_6_) *δ* 77.1 (1C, thiazole), 116.9 (1C, CH, pyridine), 127.3, 127.4, 127.5, 127.6, 128.8, 128.9, 129.1, 129.5, 129.6, 133.2, 133.4, 135.1, 137.1, 137.2, 137.8, 142.4, 142.5, 143.3, 149.4, 149.5, 149.6, 151.1, 154.7, 154.8 (28C, Ar–C), 171.9, 172.0 (2C, two imidic carbonyl groups), 176.3 (1C, C = S, group); MS (70 ev, %): *m/z* = 609 (M^+^, 100%); Anal. Calc. for C_33_H_19_N_7_O_2_S_2_ (609.68): C, 65.01(64.93); H, 3.14 (3.22); N, 16.08 (16.20).

## Synthesis of 4-amino-3-benzoyl-2-oxo-8-phenyl-10-(quinolin-2-yl)-pyrimido [4′,5′:4,5] thiazolo [2,3-*b*] pyrido[3″,2″-e] pyrimidine-11-one (11b)

The yellowish crystals were obtained by reacting compound **6** (4.46 g, 0.01 mol) and benzoyl isocyanate (1.30 mL, 0.01 mol). The compound was crystallized from DMF, with an (70%) yield, and a melting point > 350 °C (dec.); IR (*ν*, cm^−1^) KBr: 3420–3415 (NH_2_), 3096 (CH aryl), 2985(CH alkyl),1700, 1692, 1683 (3CO, three imidic carbonyl group), 1634(C = N), 1590 (C = C); ^1^H NMR (DMSO-*d6*, ppm) *δ* 6.80 a broad singlet signal (br.s, NH_2,_ D_2_O exchangeable), 7.02–7.98 (m, 16H, two phenyl, quinoline), 8.25 (s, 1H, pyridine); ^13^C NMR (DMSO-*d*_6_) *δ* 75.5 (1C, thiazole), 115.8 (1C, CH, pyridine), 118.4, 119.2, 121.3, 125.2, 126.9, 127.2, 127.6, 127.8, 128.2, 128.9, 129.1, 130.2, 131.5, 132.4, 137.2, 139.3, 144.4, 150.3, 150.6, 152.5, 154.2, 155.1, 156.4, 158.5 (28C, Ar–C), 160.2, 165.5, 170.1(3C, three imidic carbonyl groups); MS (70 ev, %): *m/z* = 593 (M^+^, 92%); Anal. Calc. for C_33_H_19_N_7_O_3_S (593.62): C, 66.77(66.85); H, 3.23(3.18); N, 16.52 (16.61).

## Pharmacological studies

The various human carcinoma cell lines (KB, CNE2, MGC-803, and MCF-7) were obtained from the National Cancer Institute at Cairo University, Egypt. Table 2 Categorizes and provides descriptions of all cancer cell lines.

**Table 2.** A description and explanation of all types of cancer cell lines used.

Cell line	Cellosaurus CVCL number	Claimed tissue origin	Registration: International cell line authentication committee
KB	CVCL_0372	(Human), epidermoid oral carcinoma	ICLAC-00010: ATCC = CRL-3596
CNE2	CVCL_6889	(Human), nasopharyngeal carcinoma	ICLAC-00474
MGC-803	CVCL_5334	(Human), gastric mucinous adenocarcinoma	ICLAC-00588
MCF-7	CVCL_0031	(Human), Breast cancer cell line	(ATCC); CRL-12584.

## *In vitro*: a method for cytotoxicity testing

The synthesized compounds were tested for cytotoxicity against various cancer cell lines *in vitro* using the MTT assay, as described previously (Mosmann 1983; Skehan et al. 1990). This assay is based on the reduction of soluble 3-(4,5-dimethyl-2-thiazolyl)-2,5-diphenyl-2*H*-tetrazolium bromide (MTT) into a blue-purple formazan product, primarily by mitochondrial reductase activity within living cells. Cells were cultured in RPMI 1640 medium supplemented with 10% fetal calf serum. Fetal calf serum, commonly referred to as Fetal Bovine Serum (FBS), is obtained from the blood of bovine fetuses. It is purchased from Sigma-Aldrich (R8758) as RPMI-1640 Medium for the cytotoxicity assay. The cells were suspended in the medium at a (2Y’104/mL) cells/mL density and plated into 96-well culture plates. They were then incubated at 37 °C in a 5% CO_2_ environment. After 12 h, 2 μL of the test sample was added to the cells in the 96-well plates, and the cultures were maintained at 37 °C for an additional 3 days. Following incubation, 20 μL of MTT solution was added to each well, and the plates were incubated for 4 h at 37 °C. After this incubation, the supernatant was carefully removed from each well, and 100 μL of DMSO was added to dissolve the formazan crystals formed during the cellular reduction of MTT. The contents of each well were mixed using a mechanical plate mixer, and the absorbance was measured with a microplate reader at a test wavelength of 570 nm. The results were expressed as the IC_50_, which represents the drug concentration that induces a 50% inhibition of cell growth compared to the control cells. Each experiment was conducted at least three times, and there was good reproducibility among replicate wells, with standard errors below 10%. The results are summarized in Table 3.

**Table 3.** Targets of anticancer proteins, PDB IDs, active site coordinates, and reference ligands.

Assay	Protein Targets	PDB ID	Resolutions	Active site coordinates:			Reference	Nativ ligand	Affinity (Kcal.mol-1)	RMSD Values
				X	Y	Z				
**Anticancer Activity**	Epidermal Growth Factor Receptor	**1M17**	2.60 Å	19.96	5.08	20.12	5-fluorouracil	AQ4	−8.50	2.20
	Cyclin-dependent kinase 2	**2A4L**	2.40 Å	27.52	63.35	−2.29	5-fluorouracil	RRC	−8.10	1.95
	Estrogen Receptor (ER)	**3ERT**	1.90 Å	72.07	36.33	11.54	5-fluorouracil	OHT	−7.50	2.40
	Vascular endothelial growth factor receptors	**3WZE**	1.90 Å	22.45	5.22	43.22	5-fluorouracil	BAX	−9.20	1.75

## Computational methods

### Molecular docking of synthesized compounds

All protein receptors were obtained from the RCSB database, as listed in Table 3. The target protein structures were preprocessed using PyMOL software, which involved removing water molecules, ions, and existing ligands. The structures of the compounds were created using BIOVIA Draw. These compounds were then converted into the mol2 format with Open Babel (O’Boyle et al. 2011). After that, AutoDock tools were utilized to convert the molecules into the pdbqt format. Before docking, ligand-centered maps were generated using AutoDock Vina (Eberhardt et al. 2021). Finally, the Discovery Studio program analyzed the 2-D interactions between the targets and the ligands.

### In-silico pharmacokinetics, ADME, and toxicity prediction

The compounds’ physicochemical parameters and ADMET were analyzed using BIOVIA Discovery Studio software (BIOVIA 2017).

### Molecular dynamics (MD) simulation

Molecular dynamics (MD) simulations are widely used to investigate the binding interactions and affinities of protein-ligand complexes. In this study, we conducted molecular dynamics (MD) simulations using the GROMACS 2018 software to validate the reliability of our docking results further. We generated the protein topology using CHARMM36 force field parameters, while the topologies for the ligands were created using the Geoff server. The ligands were subjected to positional constraints. We performed NVT (constant number of particles, volume, and temperature) and NPT (constant number of particles, pressure, and temperature) equilibrations for 1,000 picoseconds (ps) at a temperature of 300 K and a pressure of 1.0 bar. Following the MD simulations, we calculated the Root Mean Square Deviation (RMSD), Root Mean Square Fluctuation (RMSF), and radius of gyration (Rg) (Páll et al. 2020).

## Results

### Synthesis

**Scheme 1.**
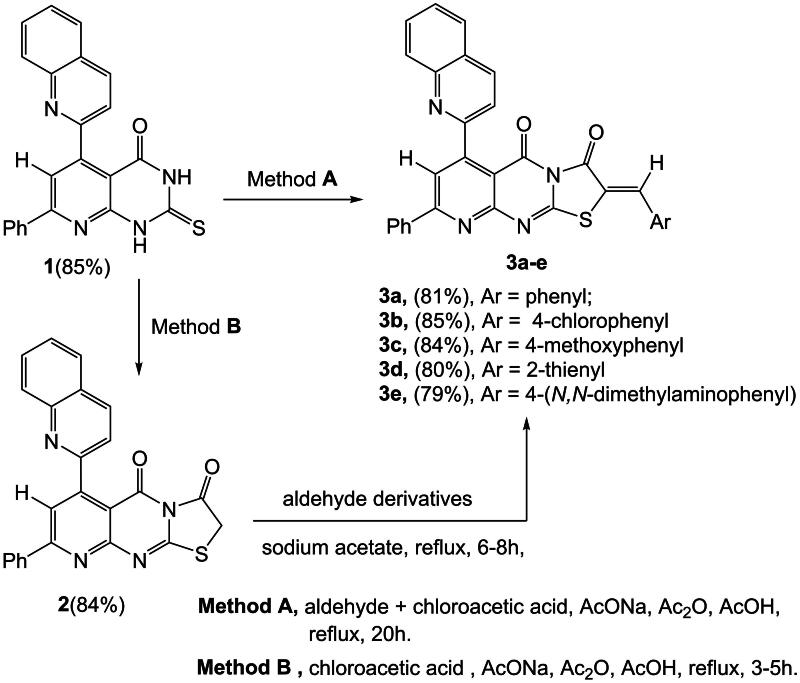
Synthesis of 2-substituted-8-phenyl-6-(quinolin-2-yl)-pyrido[2,3-*d*] thiazolo[3,2-*a*] pyrimidines **(3a–e)**.

Method A: A one-pot synthesis involves heating a ternary mixture of 7-phenyl-5-(quinolin-2-yl)-2-thioxo-2,3-dihydropyrido[2,3-*d*] pyrimidin-4(1*H*)-one **(1)** *via* (Abu-Hashem et al. 2023, 2024; Abu-Hashem and Al-Hussain 2024), chloroacetic acid, and a proper aldehyde under reflux in acetic acid and acetic anhydride, along with anhydrous sodium acetate, to yield 2-substituted-8-phenyl-6-(quinolin-2-yl)-5*H*-pyrido[2,3-*d*] thiazolo[3,2-*a*] pyrimidine-3,5 (2*H*)-dione **(3a–e)** in high yields. Alternatively, compounds **(3a–e)** can be synthesized by reacting compound **(2)** with aromatic aldehyde derivatives. Method B: This method involves refluxing a mixture of compound **(1)** and chloroacetic acid in acetic acid and acetic anhydride, along with anhydrous sodium acetate, to produce 8-phenyl-6-(quinolin-2-yl)-5*H*-pyrido[2,3-*d*] thiazolo [3,2-a] pyrimidine-3,5(2*H*)-dione **(2)** in good yields. The IR spectrum of the compound **(2)** established absorption bands at *ν* 1691, 1685 cm^−1^, conforming to two imidic carbonyl groups. The ^1^H-NMR spectrum of (**2)** showed two singlets at *δ* 3.90, 8.15 ppm corresponding to two protons of CH_2_, the thiazole ring and one proton of the pyridine ring. The ^1^H-NMR spectrum of (**3a)** exposed two singlets at *δ* 8.20 and 8.30 ppm, corresponding to one methine proton and one proton of the pyridine ring. The mass spectra of compounds **3a–e** exhibited a molecular ion peak that represents a positively charged radical cation at the following m/z values: 510 (M^+^, 100%), 545 (M^+^, 100%), 540 (M^+^, 100%), 516 (M^+^, 100%), and 553 (M^+^, 90%). The elemental analysis and the spectroscopic data from IR, NMR, and MS are detailed in the experimental section, and the chemical reactions of compounds are illustrated in Scheme 1.

**Scheme 2.**
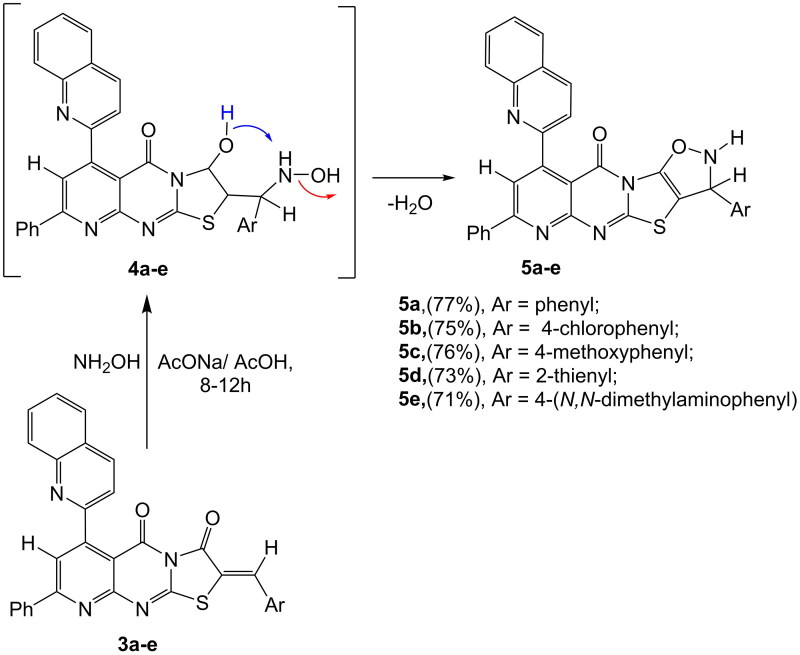
3-(Substituted)-9-(quinolin-2-yl)-10*H*-isoxazolo [5′,4′:4,5] thiazolo [3,2-*a*] pyrido[2,3-*d*] pyrimidinone **(5a–e)**.

The formation of compounds **(5a–e)** from **(3a–e)** begins with an initial 1,4-addition of hydroxylamine to the ethylenic double bond, resulting in the intermediate compound **(4a–e)**, which subsequently undergoes a loss of water. Compounds **(3a–e)** can also react with hydroxylamine hydrochloride through cycloaddition, achieved by boiling acetic acid with anhydrous sodium acetate. This process leads to the formation of 3-(substituted)-7-phenyl-9-(quinolin-2-yl)-2,3-dihydro-10*H*-isoxazolo [5′,4′:4,5] thiazolo [3,2-*a*] pyrido [2,3-*d*] pyrimidin-10-one **(5a–e)**. The IR spectrum of compound **(5a)** displays absorption bands at *ν* 3310 cm^−1^, corresponding to the (NH) group, and at 1684 cm^−1^, indicative of an imidic carbonyl group. The ^1^H-NMR spectrum of **(5a)** features a singlet at *δ* 9.90 ppm, confirming the presence of one proton from the (NH) group exchangeable with D_2_O. Furthermore, the ^13^C-NMR spectrum of **(5a)** reveals absorption peaks at *δ* 63.8 and 64.4 ppm, indicating two carbon atoms in the isoxazole ring, and at *δ* 165.7 ppm, confirming one carbon atom from the carbonyl group, as illustrated in Scheme 2.

**Scheme 3.**
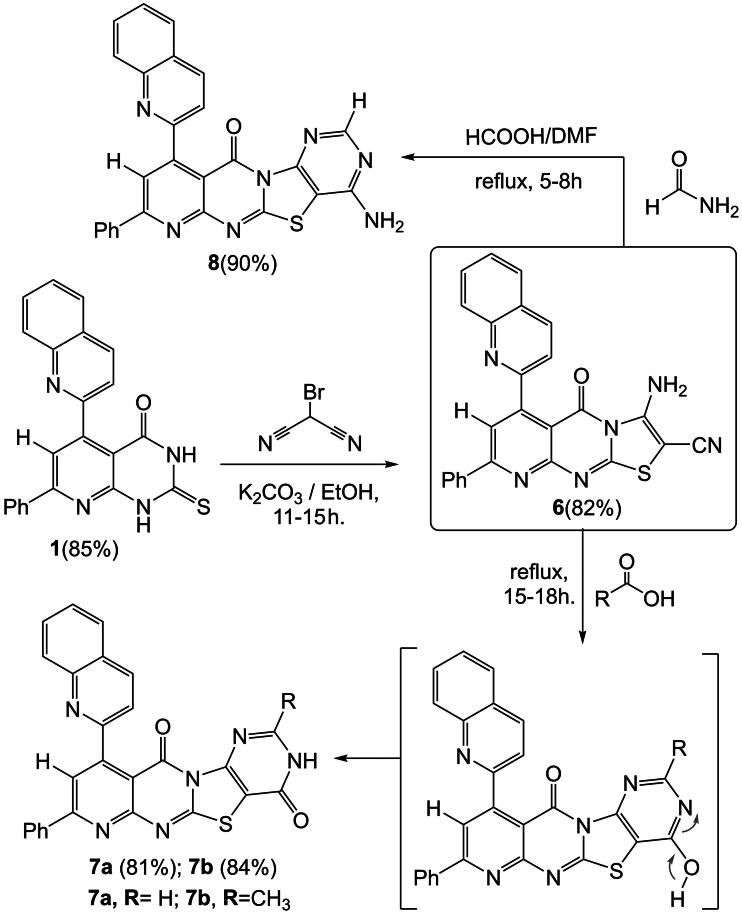
8-Phenyl-10-(quinolin-2-yl)-pyrimidothiazolo[2,3-*b*] pyridopyrimidinone derivatives.

This study was designed to synthesized poly-heterocyclic compounds possessing 6-quinoline-pyrido[2,3-*d*] thiazolo[3,2-*a*] pyrimidine-2-carbonitrile nucleus, which could be used as a precursor for the synthesis of a series of new compounds having new ring systems such as substituted-10-(quinoline)-pyrimidothiazolo[2,3-*b*] pyridopyrimidinone derivatives. Thus, the reaction of 7-phenyl-5-(quinolin-2-yl)-2-thioxo-2,3-dihydropyrido[2,3-*d*] pyrimidinone **(1)** with bromomalono-nitrile in an aqueous alcoholic potassium carbonate solution gave 3-amino-5-oxo-8-phenyl-6-(quinolin-2-yl)-5*H*-pyrido[2,3-*d*] thiazolo [3,2-*a*] pyrimidine-2-carbonitrile **(6)**. The IR spectrum compound **(6)** exhibited absorption bands at *ν* 3420 cm^−1^ conforming to one (NH_2_) group, 2215 cm^−1^ corresponding to one carbonitrile group, and 1692 cm^−1^ corresponding to one imidic carbonyl group. Additionally, the ^1^H NMR spectrum of (6) displayed a single singlet at *δ* 9.90 ppm, a broad singlet signal, corresponding to one (NH_2_) group and two exchangeable protons with D_2_O. Compound (**6),** as a typical *β*-enamino-nitrile derivative, reacted with aliphatic acids, namely, formic acid and acetic acid, to produce Substituted-8-phenyl-10-(quinolin-2-yl)-pyrimido[4′, 5′: 4, 5] thiazolo [2, 3-*b*] pyrido[3″, 2″-*e*] pyrimidine-4,11(3*H*)-dione **(7a, b)**. Similarly, compound (**6)** reacted with formamide, in the presence of formic acid and dimethylformamide, to yield 4-amino-8-phenyl-10-(quinolin-2-yl)-pyrimido [4′, 5′: 4,5] thiazolo[2,3-b] pyrido [3″, 2″- e] pyrimidine-11-one **(8)**. The IR spectrum of **(7a)** was determined, revealing a broad (NH) group at ν 3250–3260 cm^ − 1^ and two carbonyl groups at around *ν* 1685–1680 cm^−1^. The ^1^H-NMR spectrum of **(7a)** showed a detected singlet at *δ* 9.00 ppm, representing the presence of one proton of the (NH) group, which is exchangeable with (D_2_O). The infrared (IR) spectrum of compound **(8)** displayed absorption bands at wave numbers of *ν* 3410 cm^−1^, which correspond to an amine group (NH_2_), and at *ν* 1677 cm^−1^, which corresponds to a carbonyl group. Additionally, the proton nuclear magnetic resonance (^1^H-NMR) spectrum of compound **(8)** exhibited a singlet signal at a chemical shift of *δ* 6.30 ppm. This is a broad singlet signal that corresponds to the two protons of the amino group (NH_2_) and is exchangeable with deuterium oxide (D_2_O), as shown in Scheme 3.

**Scheme 4.**
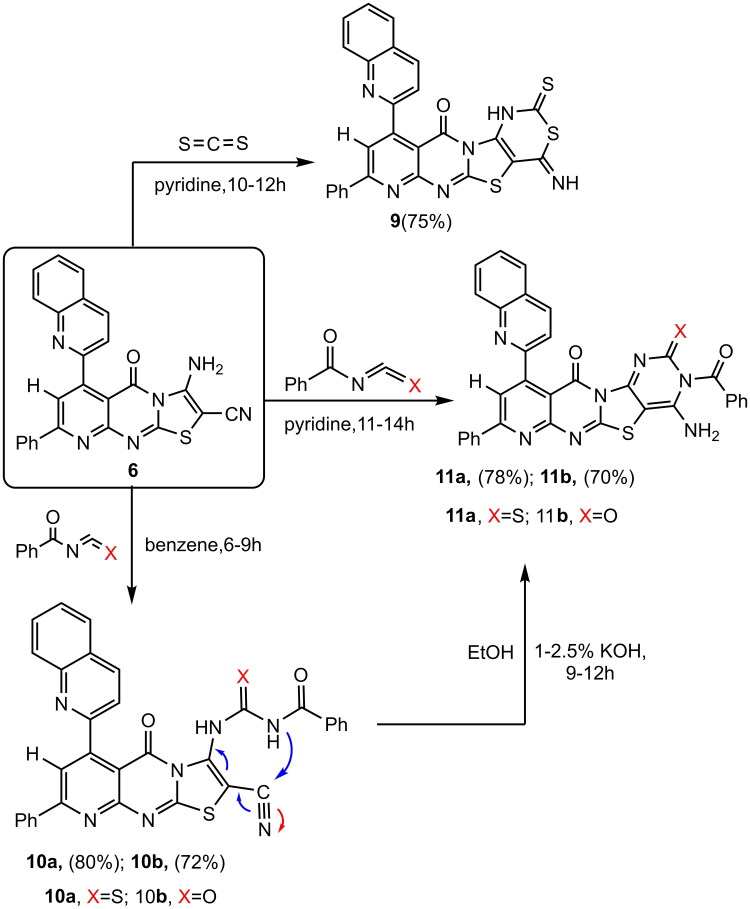
4-Amino-3-benzoyl-2-Sub-10-(quinoline)-pyrimidothiazolopyridopyrimidinone derivatives.

In contrast, according to the method developed, when compound **(6)** was heated with carbon disulfide in pyridine, 4-imino-8-phenyl-10-(quinolin-2-yl)-2-thioxo-1,4-dihydro-2*H*, 11 *H*-pyrido [2″, 3″: 4′,5′] pyrimido [2′,1′:2,3] thiazolo[4,5-*d*] [1,3] thiazin-11-one **(9),** was produced. In the IR spectrum of compound **(9)**, broad bands were observed at *ν* 3290–3270 cm^−1^, indicating the presence of the (2NH) group. Additionally, two sharp peaks at ν 1673 cm^−1^ and 1352 cm^−1^ corresponded to the carbonyl and (C = S) groups, respectively. The ^1^H-NMR spectrum of compound **(9)** displayed two singlet signals at *δ* 9.10 and 9.90 ppm, representing the two protons of the (2NH) groups. To synthesize **(11a, b)** derivatives of condensed compound aminonitriles **(6)**, we developed a method consisting of the reaction of the aminonitriles **(6)** with benzoyl-iso(thio)cyanates in refluxing of pyridine as base long times 11-14hours followed by cyclisation of the 4-amino-3-benzoyl-2-substituted-8-phenyl-10-(quinolin-2-yl)-pyrimido[4′,5′:4,5] thiazolo [2,3-*b*]pyrido[3″, 2″-*e*] pyrimidine-11-one **(11a, 11b)** derivatives. Another procedure, reaction of the aminonitriles **(6)** with benzoyl-iso (thio) cynate, in refluxing dry benzene to give the resulting compounds *N*-((2-cyano-5-oxo-8-phenyl-6-(quinolin-2-yl)-5*H*-pyrido[2,3-*d*]thiazolo [3, 2*-a*] pyrimidin-3-yl) carbamothioyl or carbamoyl) benzamide **(10a, b)** derivatives by the action of a base afforded compounds **(11a, b)**, When a 1.5% aqueous solution of potassium hydroxide or 1-2.5% solution of potassium hydroxide in ethanol is used, are formed **(11a, b)** (Paronikyan et al. 1993; Ghorab et al. 2011). The IR spectrum of compound **(10a)** displayed a broad band at wavenumbers of 3310, 3200, 2230, 1695, 1684, and 1200 cm^−1^. These bands correspond to two amine groups (2NH), one carbonitrile group, two carbonyl groups, and a thioketone group (C = S). The ^1^H NMR spectrum of compound **(10a)** revealed two singlet signals at *δ* 11.03 and 12.20 ppm; these signals originated from two exchangeable proton sets (2NH) and were exchangeable with D_2_O. The IR spectrum of compound **(11a)** exhibited a broad band at *ν* 3415 cm^−1^, corresponding to one (NH_2_) group. Additionally, it displayed bands at 1698, 1690, and 1210 cm^−1^, which are attributed to two carbonyl groups and a (C = S) group, respectively. The ^1^H-NMR spectrum of compound **(11a)** revealed a broad singlet signal at *δ* 6.78 ppm, indicating the presence of two protons from the (NH_2_) group, which are exchangeable with D_2_O. The molecular ion peak represents a positively charged radical cation for compounds **9**, **10a**, **10b**, **11a**, and **11b** identified in the mass spectrometry analysis were observed at the following m/z values: 522 (M^+^, 100%), 609 (M^+^, 99%), 593 (M^+^, 95%), 609 (M^+^, 100%), and 593 (M^+^, 92%). Detailed spectral data for these new compounds are presented in the Experimental Section, as illustrated in Scheme 4.

### Pharmacology evaluation (In vitro cytotoxicity)

The results indicated that incorporating pyrimidothiazole, isoxazole, and thiazole moieties into the structure improved anticancer activity. In contrast, compounds that did not contain these moieties, specifically pyrido[2,3-*d*] pyrimidines, exhibited weak effects against tumour cells. We applied the conventional MTT (3-[4,5-dimethyl-2-thiazolyl)-2,5-diphenyl-2*H*-tetrazolium bromide) technique to achieve this assessment (Mosmann 1983; Skehan et al. 1990). Some derivatives of quinoline-pyridopyrimidines, including isoxazolothiazolopyrido[2,3-*d*] pyrimidinones **(5a–e)**, pyrido-thiazolo[3,2-*a*] pyrimidine (carbamothioyl or carbamoyl) benzamide **(10a–b)**, and pyrimido-thiazolo[2,3-*b*] pyridopyrimidinone **(11a–b)**, demonstrated significant cytotoxicity against various cancer cell lines. These cell lines included drug-sensitive human oral carcinoma cells (KB), human nasopharyngeal carcinoma cells (CNE2), human gastric carcinoma cells (referred to as MGC-803), and human breast adenocarcinoma cells (MCF-7). Compounds **6, 7a–b, 8,** and **9** also displayed moderate cytotoxicity against the same cancer cell lines. In contrast, compounds **1, 2**, and **3a–e** exhibited weaker cytotoxicity, as indicated by their respective IC_50_ values in Table 4.

**Table 4.** The cytotoxic effects of quinoline-pyridothiazolopyrimidines on human carcinoma cell lines.

*In vitro* cytotoxicity IC_50_(µM)				Compounds
MCF-7^a^	MGC-803^a^	CNE2^a^	KB^a^	
>50	>50	>50	49.5 ± 1.6	**1**
49.1 ± 1.1	48.2 ± 2.3	47.9 ± 2.2	47.5 ± 1.3	**2**
46.1 ± 2.4	45.8 ± 2.4	44.5 ± 2.7	44.2 ± 1.5	**3a**
41.8 ± 1.6	41.5 ± 2.4	41.1 ± 2.5	40.5 ± 1.8	**3b**
43.8 ± 1.9	43.1 ± 2.2	42.7 ± 2.1	42.3 ± 1.4	**3c**
36.2 ± 2.2	35.9 ± 2.1	35.6 ± 1.7	35.1 ± 1.2	**3d**
29.9 ± 2.7	29.1 ± 2.4	29.5 ± 2.8	28.3 ± 2.7	**3e**
12.1 ± 1.7	11.4 ± 1.5	11.9 ± 1.1	11.8 ± 1.6	**5a**
11.7 ± 1.3	10.9 ± 1.1	11.2 ± 1.7	10.5 ± 1.3	**5b**
11.9 ± 1.2	11.1 ± 1.8	11.6 ± 1.4	11.3 ± 1.5	**5c**
11.5 ± 1.6	10.7 ± 1.3	10.8 ± 1.5	9.9 ± 1.4	**5d**
11.1 ± 1.4	10.3 ± 1.2	10.7 ± 1.3	9.8 ± 1.1	**5e**
22.8 ± 2.6	22.5 ± 2.3	21.4 ± 2.1	20.1 ± 2.5	**6**
16.5 ± 2.4	16.3 ± 1.7	15.6 ± 1.8	15.3 ± 2.4	**7a**
16.1 ± 2.2	15.9 ± 1.3	15.4 ± 1.5	15.2 ± 2.1	**7b**
14.8 ± 1.4	14.4 ± 1.2	14.5 ± 1.7	14.1 ± 1.9	**8**
13.9 ± 1.5	13.6 ± 1.8	13.8 ± 1.3	13.5 ± 1.2	**9**
12.5 ± 1.1	11.8 ± 1.4	12.7 ± 1.9	12.4 ± 1.5	**10a**
13.4 ± 1.3	12.1 ± 1.6	13.1 ± 1.1	12.8 ± 1.2	**10b**
10.6 ± 0.5	9.5 ± 0.8	10.2 ± 0.9	9.1 ± 0.7	**11a**
10.8 ± 0.9	9.9 ± 1.1	10.5 ± 0.7	9.4 ± 0.6	**11b**
13.1 ± 1.4	11.7 ± 1.2	12.4 ± 1.3	11.5 ± 1.1	**5-Fluorouracil**

The KB cells were drug-sensitive human oral carcinoma cells. The CNE2 cells were drug-sensitive human nasopharyngeal carcinoma cells. The MGC-803 cells were drug-sensitive human gastric carcinoma cells. The MCF-7 cells were human breast adenocarcinoma cells.

### Study of structure-activity relationship (SAR)

Subsequently, analyzing the data presented in Table 4, we calculated the therapeutic index (TI), which is the ratio of the IC_50_ (the concentration required to inhibit 50% of cells) in normal cells to that in cancer cells. Our analysis concluded that products **11a–b, 5a–e**, and **10a–b** are the most promising and effective potential anticancer drugs in that order. These products demonstrate a significantly better therapeutic index (TI) than the standard drug, 5-fluorouracil. Chemotherapy is a treatment that uses powerful medications to target and destroy rapidly growing cells in the body. It primarily treats cancer, as cancerous cells grow and replicate significantly faster than normal cells.

Our search for chemotherapy drugs identified several medications used alone or in combination to treat various types of cancer cells. In this manuscript, we present our current study, which reveals a significant similarity in the chemical composition between these drugs and the compounds we recently synthesized. We discuss the synthesized compounds’ molecular structure and functional groups and compare them with standard medications and findings from previous scientific studies on cancer treatment. The results of our study are outlined as follows:

- The broad requirement for cytotoxicity activities may stem from the diversity of structures in new heterocyclic compounds, including quinolines, pyrimidothiazolo[2,3-*b*] pyridopyrimidines, pyrido[2,3-*d*] pyrimidinones, isoxazolothiazolo[3,2-*a*] pyrido[2,3-*d*] pyrimidinones and *N*-((2-cyano-6-(quinoline)-pyrido[2,3-*d*] thiazolo[3,2-*a*] pyrimidine) carbamothioyl or carbamoyl) benzamide.
- Several compounds have shown excellent efficacy against cancer cell lines in vitro, including substituted quinolines, substituted-pyrimido[4′, 5′: 4, 5] thiazolo [2, 3-*b*] pyrido [3″, 2″- e] pyrimidine-11-one **(11a–b)**, substituted-isoxazolo[5′,4′:4,5]thiazolo[3,2-*a*]pyrido[2,3-*d*] pyrimidin -10-one **(5a–e)**, and *N*-((2-cyanopyridothiazolo[3,2-*a*] pyrimidine) carbamothioyl/carbamoyl) benzamide **(10a–b)**. Previous scientific studies support these findings. These compounds exhibit a variety of properties, including cytotoxic activities, which can be attributed to their diverse functional groups and moieties such as pyridine, quinoline, pyrimidine, thiazole, isoxazole, benzoyl, phenyl, 4-chlorophenyl, 4-methoxyphenyl, 2-thienyl, 4- (*N, N*-dimethyl-amino-phenyl), amino, methyl and carbonitrile groups. They also incorporate several heteroatoms, including sulfur, nitrogen, and oxygen. Previous research has indicated that some similar compounds exhibit significant biological activity as anticancer agents (Beyer et al. 2008; Farghaly and Abdalla 2009; Riyadh et al. 2010; Abu-Hashem et al. 2011, Abu-Hashem and El-Shazly 2017; Ghorab et al. 2011; Abu-Hashem & Aly 2012, Abu-Hashem et al. 2010; Abu-Hashem & Badria 2015; Abu-Hashem & Hussein 2015; Gu et al. 2017; Wang et al. 2017; Abdelbaset et al. 2018; Costa et al. 2020; Gouda et al. 2020; Mohamed and Abuo-Rahma 2020; Abd El-Sattar et al. 2021; Khajouei et al. 2021; Abu-Hashem & Al-Hussain 2022; Albratty and Alhazmi 2022; Al-Warhi et al. 2022; Nossier et al. 2022; Dos Santos Correia et al. 2023; Abu-Hashem et al. 2024, 2021a, 2021b, 2020; Aghahosseini et al. 2024; Ali et al. 2024; Arzine et al. 2024; Boutalaka et al. 2024; Deshpande et al. 2024; Kumar et al. 2024; Mokbel et al. 2024; Sireesha et al. 2024; Bufarwa et al. 2025).
- Subsequently, the products **11a–b**, **5a–e** and **10a–b** are highly effective in treating specific carcinoma cell lines, particularly human oral carcinoma cells **(KB)**, human nasopharyngeal carcinoma cells (**CNE2)**, human gastric carcinoma cells **(MGC-803)** and human breast adenocarcinoma cells **(MCF-7)**. These compounds contain various functional groups and moiety derivatives, as illustrated in Schemes 1–4 and Table 4, which contribute to their superior cytotoxicity compared to the standard drug, 5-Fluorouracil.

## Discussion

### Computational analysis

#### Docking and molecular interaction of synthesized compounds

**Figure 4.**
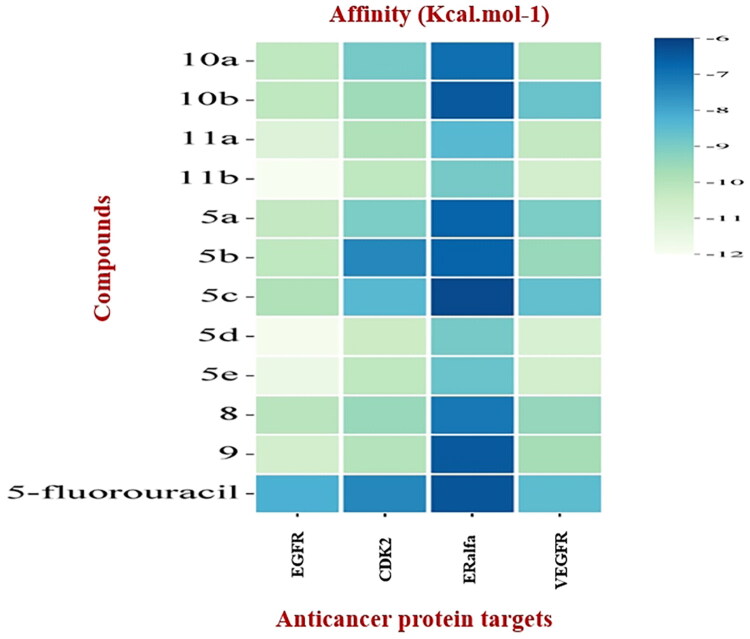
Heatmap of binding affinity of compounds with the targets of proteins.

Given the significance of prior molecular docking studies involving various compounds and their interactions with proteins (El-Helw et al. 2024, 2024; El-Sewedy et al. 2025), this study has focused on how synthetic new compounds interact with protein targets that are associated with anticancer properties. This analysis aimed to provide insights into the effectiveness of these compounds. The results of the docking experiments, presented in Table 5 and Figure 4, illustrate the binding affinities between the compounds and four anticancer receptors.

**Table 5.** Binding affinity of ligands with targets of anticancer activity.

No.	Ligands	Affinity (Kcal.mol-1)			
		*EGFR*	*CDK2*	*ERalfa*	*VEGFR*
		(PDB.ID: 1M17)	(PDB.ID: 2A4L)	(PDB.ID: 3ERT)	(PDB.ID: 3WZE)
**1**	**10a**	−10.2	−8.9	−6.9	−10
**2**	**10b**	−10.2	−9.6	−6.5	−8.7
**3**	**11a**	−11.1	−9.9	−8.4	−10.3
**4**	**11b**	−12	−10.2	−8.9	−10.7
**5**	**5a**	−10.3	−9	−6.7	−9
**6**	**5b**	−10.2	−7.4	−6.7	−9.5
**7**	**5c**	−9.9	−8.4	−6.2	−8.6
**8**	**5d**	−11.9	−10.5	−8.9	−10.9
**9**	**5e**	−11.6	−10.2	−8.7	−10.7
**10**	**8**	−10.1	−9.5	−7.1	−9.4
**11**	**9**	−10.7	−10	−6.5	−9.7
**12**	**5-fluorouracil**	−8.2	−7.4	−6.4	−8.5

#### Docking and molecular interaction with epidermal growth factor receptor (EGFR)

**Figure 5.**
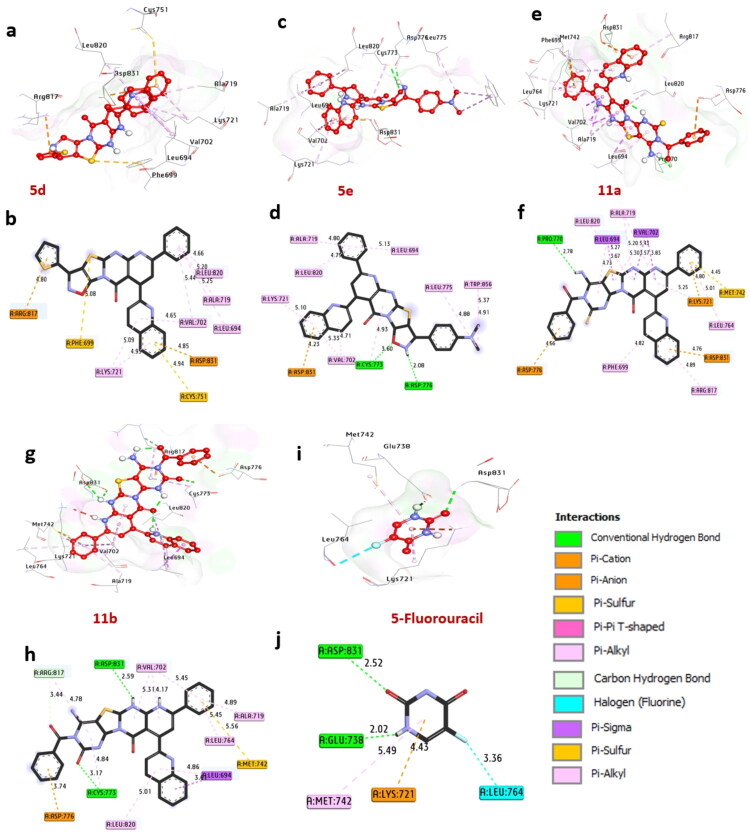
3D Representations of compound conformations at the binding pocket of EGFR (PDB: ID 1M17): (A and B) **5d**, (C and D) **5e**, (e and f) **11a**, (G and H) **11b**, and (I and J) 5-fluorouracil.

EGFR, or epidermal growth factor receptor, is a cell surface receptor that plays a crucial role in regulating cell growth, survival, and differentiation. The significance of this receptor is particularly notable in triple-negative breast cancer (TNBC). Docking analysis has demonstrated that the compounds **5d**, **5e**, **11a**, and **11b** show a substantial affinity for EGFR, with binding energies of −11.90, −11.60, −11.10, and −12.00 kcal/mol, respectively. These values exceed 5-fluorouracil, which has a binding energy of −8.20 kcal/mol. Compounds **5e**, **11a**, and **11b** form hydrogen bonds with essential amino acids, including Asp776, Cys773, Pro770, and Asp831. Additionally, they engage in non-hydrophilic interactions with various amino acids, including Pi-alkyl interactions with Leu820, Ala719, Leu694, Val702, Lys721, Trp856, and Leu775. The binding interactions also include Pi-Sigma interactions with Leu694 and Val702, Pi-Cation interactions with Lys721, Asp776, Asp831, and Arg817, as well as sulfur interactions with Cys751, Phe699, and Met742. Key amino acids in the catalytic site, such as Asp831, Cys773, Pro770, and Phe699, contribute to the enhanced binding affinity of these compounds. These findings suggest that compounds **5d**, **5e**, **11a**, and **11b** are promising inhibitors of EGFR, warranting further investigation (see Figure 5, Table 6). This aligns with previous research (Rashdan et al. 2021), which identified EGFR as an anticancer protein receptor explored for its anti-inflammatory properties through molecular docking analysis. Furthermore, studies (Al-Rashood et al. 2020; Bawazir et al. 2025) employed docking techniques to examine the inhibitory interactions of small compounds against EGFRWT, along with references EGFRL858R and EGFRT790 M.

**Table 6.** Molecular interactions of ligands with amino acids of EGFR (PDB: ID 1M17).

No.	Protein	Ligand	Hydrophilic Interactions	Hydrophobic Contacts			No. of H-Bonds	No. of Total Bonds	Affinity kcal mol^-1^
			Residue (H-Bond)	Length	Residue (Bond type)	Length			
1	**EGFR (PDB: ID 1M17)**	**5d**	–	–	Leu820, (Pi-alkyl) Ala719, (Pi-alkyl) Leu694, (Pi-alkyl) Val702, (Pi-alkyl) Lys721, (Pi-alkyl) Val702, (Pi-alkyl) Asp831, (Pi-Cation) Arg817, (Pi-Cation) Cyss751, (Sulfur) Phe699, (Sulfur)	4.66 5.20 5.25 5.44 5.09 4.65 4.85 4.80 5.08	**0**	**10**	**−11.90**
2		**5e**	Asp776 (H-Bond) Cys773 (H- Bond)	2.08 3.60	Trp856, (Pi-alkyl) Leu775, (Pi-alkyl) Leu694, (Pi-alkyl) Ala719, (Pi-alkyl) Leu820, (Pi-alkyl) Lys721, (Pi-alkyl) Val702, (Pi-alkyl) Val702, (Pi-alkyl) Asp831, (Pi-Cation)	5.37 4.88 5.13 4.80 4.79 5.10 4.71 5.33 4.23	**2**	**11**	**−11.60**
3		**11a**	Pro770 (H-Bond)	2.78	Leu820, (Pi-alkyl) Ala719, (Pi-alkyl) Leu764, (Pi-alkyl) Arg817, (Pi-alkyl) Phe699, (Pi-alkyl) Leu694, (Pi-Sigma) Val702, (Pi-Sigma) Lys721, (Pi-Cation) Asp831, (Pi-Cation) Asp776, (Pi-Cation) Met742, (Sulfur)	5.27 5.20 5.01 4.89 4.82 5.27 3.27 4.80 4.76 4.66 4.45	**1**	**11**	**−11.10**
4		**11b**	Asp831 (H-Bond) Cys773, (H- Bond)	2.59 3.17	Val702, (Pi-alkyl) Val702, (Pi-alkyl) Leu820, (Pi-alkyl) Leu764, (Pi-alkyl) Ala719, (Pi-alkyl) Leu694, (Pi-sigma) Asp776, (Pi-Cation) Met742, (Sulfur)	5.45 5.31 5.01 5.45 4.89 3.41 3.74 5.56	**2**	**10**	**−12.00**
5		**5-fluorouracil**	Asp831 (H-Bond) Glu738 (H-Bond)	2.52 2.02	Met742, (Pi-alkyl) Val702, (Pi-Cation) Leu764, (halogen)	5.49 4.43 3.36	**2**	**5**	**−8.20**

#### Docking and interaction with Cyclin-dependent kinase 2(CDK2)

**Figure 6.**
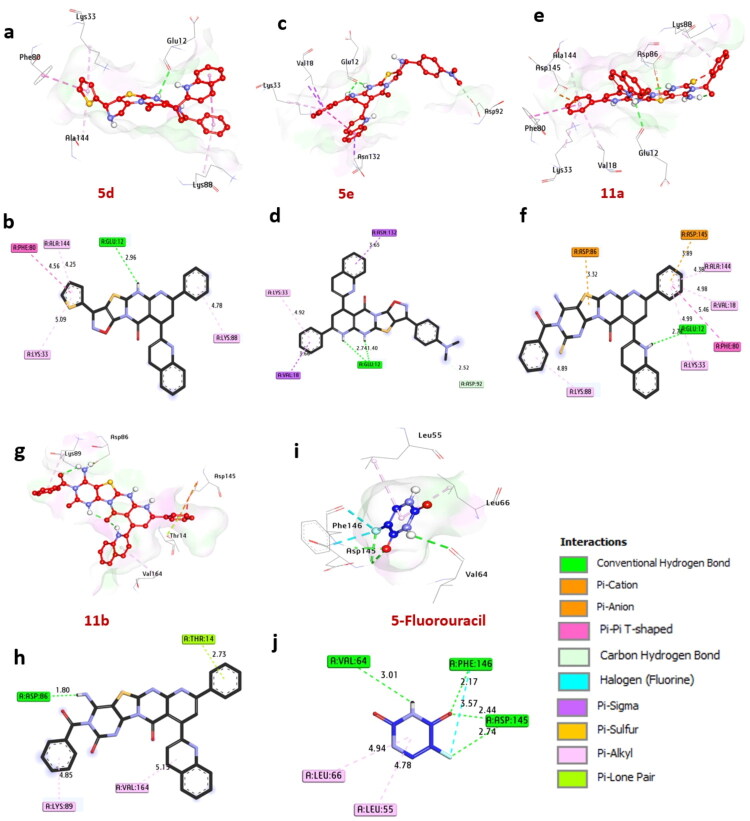
3D Representations of compounds conformations at the binding pocket of Cyclin-dependent kinase 2 (PDB:ID 2A4L): (A and B) **5d**, (C and D) **5e**, (E and F) **11a**, (G and H) **11b**, and (I and J) 5-fluorouracil.

Cyclin-dependent kinase 2 (CDK2) is an enzyme encoded by the CDK2 gene in humans. It belongs to the cyclin-dependent kinase family of serine/threonine protein kinases. It is critical in regulating the cell cycle, especially during the G1 to S phase transition when cells prepare for DNA replication and mitosis. In docking studies, compounds **5d**, **5e**, **11a**, and **11b** exhibited significant binding affinity, with binding energies of −10.50, −10.20, −9.90, and −10.20 kcal/mol, respectively. The binding energies of these compounds exceed that of 5-fluorouracil, which has a binding energy of −7.40 kcal/mol. The compounds form hydrogen bonds with key amino acids, including Glu12 and Asp86. Non-hydrophilic interactions involve several other amino acids such as Lys88, Lys33, Ala144, Val18, Val164, and Leu89. These interactions include alkyl bonds with Lys88, Lys33, Ala144, Val18, and Val164; an Amid-Pi stack interaction with Phe80; Pi-sigma interactions with Asn132 and Val18; Carbon-H bonds with Asp92; Pi-cation interactions with Asp145 and Asp86; and Pi-lone pair interactions with Thr14. Amino acids Glu12, Asp86, Ala144, and Lys88 within the catalytic site have been identified as positively impacting the binding affinity of these compounds. The findings suggest that compounds **5d**, **5e**, **11a**, and **11b** show promise as potential CDK2 inhibitors, warranting further investigation (see Figure 6 and Table 7). These results are consistent with previous studies (Nemr et al. 2020; Sroor et al. 2025), which also examined compounds exhibiting favorable binding and intense interactions with receptors such as CDK2, EGFR, ERα, and VEGFR through molecular docking simulations. Additionally, a prior study (Sroor et al. 2024) employed molecular docking to clarify the binding interactions of inhibitors with CDK2 and EGFR protein targets.

**Table 7.** Molecular interactions of ligands with cyclin-dependent kinase 2 (PDB:ID 2A4L).

No.	Protein	Ligand	Hydrophilic interactions		Hydrophobic contacts		No. of H-Bonds	No. of Total Bonds	Affinity kcal mol^−1^
			Residue (H-Bond)	Length	Residue (Bond type)	Length			
1	Cyclin-dependent kinase 2 (CDK2) (PDB:ID 2A4L)	**5d**	Glu12, (H-Bond)	2.96	Lys88, (alkyl) Lys33, (alkyl) Ala144, (alkyl) Phe80, (Amid-Pi-stack)	4.78 5.09 4.25 4.56	**1**	**5**	−10.50
2		**5e**	Glu12, (H- Bond) Glu12, (H- Bond)	1.40 2.74	Lys33, (alkyl) Asn132, (Pi-sigma) Val18, (Pi-sigma) Asp92, (Carbon H bond)	4.92 3.65 3.60 2.52	**2**	**6**	−10.20
3		**11a**	Glu12, (H-Bond)	2.70	Ala144, (alkyl) Val18, (alkyl) Lys33, (alkyl) Lys88, (alkyl) Phe80, (Amid-Pi-stack) Asp145, (Pi-Cation) Asp86, (Pi-Cation)	4.38 4.98 4.99 4.89 5.46 3.89 3.32	**1**	**8**	−9.90
4		**11b**	Asp86, (H- Bond)	1.80	Val164, (alkyl) Leu89, (alkyl) Thr14, (Pi-lone pair)	5.15 4.85 2.73	**1**	**4**	−10.2
5		**5-fluorouracil**	Asp145, (H-Bond) Asp145, (H-Bond) Phe146, (H- Bond) Val64, (H-Bond)	2.44 2.74 2.17 3.01	Leu66, (alkyl) Leu55, (alkyl) Phe146, (halogen)	4.94 4.78 3.57	4	7	−7.40

#### Docking and interaction with estrogen receptor (PDB: ID 3ERT)

**Figure 7.**
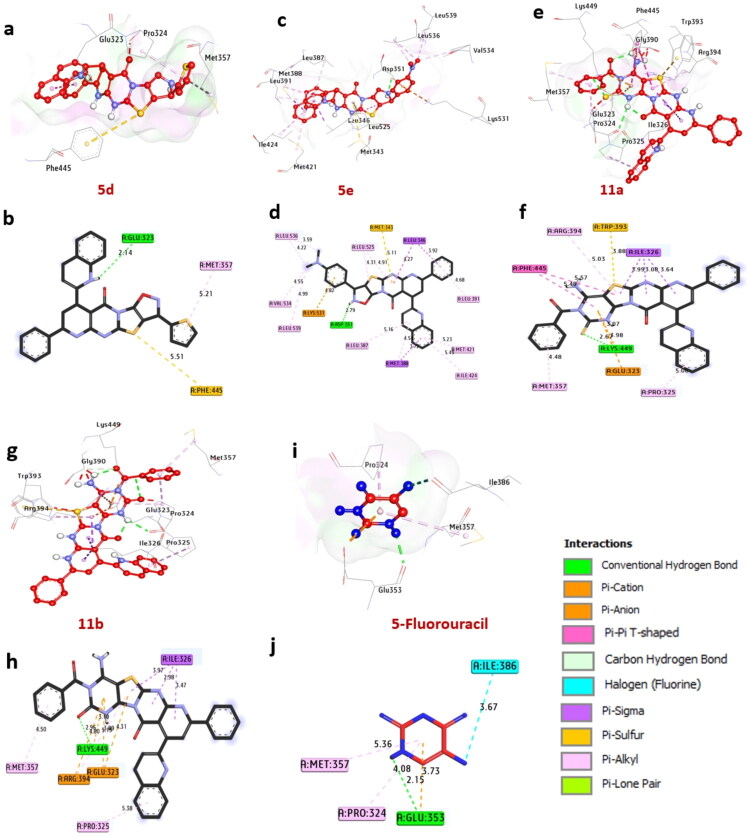
3D Representations of compounds at the binding pocket of estrogen receptor (PDB:ID 3ERT): (A and B) **5d**, (C and D) **5e**, (E and F) **11a,** (G and H) **11b**, and (I and J) 5-fluorouracil.

The Estrogen Receptor (ER) is a crucial nuclear receptor that significantly regulates the growth and proliferation of hormone receptor-positive breast cancer cells. According to the docking results, the compounds **5d**, **5e**, **11a**, and **11b** demonstrated strong binding affinities, with binding energies of −8.50, −8.90, −8.40, and −8.90 kcal/mol, respectively. These values are significantly lower than 5-fluorouracil, with a binding energy of −6.40 kcal/mol. These promising compounds formed hydrogen bonds with key amino acids, including Glu232, Asp351, and Lys449. Additionally, they exhibited various non-hydrophilic interactions with other amino acids such as Met357, Leu391, Leu525, Met421, Ile424, Leu387, Leu536, Val534, Leu539, Pro325, and Arg394. Notable interactions observed included alkyl bonds with several residues, sulfur interactions with Met343, amid-pi stacking with Phe445, pi-sigma interactions with Met388 and Ile326, and pi-cation interactions with Arg394, Glu323, and Lys531. Residues within the catalytic site, such as Glu232, Asp351, Lys449, and Arg394, have been identified as positively influencing the binding affinity of these compounds. Overall, the findings indicate that compounds **5d**, **5e**, **11a**, and **11b** are the most promising candidates for further investigation as potential inhibitors of estrogen receptors. These results are consistent with the research conducted by references (El-Khouly et al. 2022; Elmorsy et al. 2022; Khaled et al. 2023). They utilized compounds that demonstrated favorable binding in in-silico molecular docking simulations against VEGFR-2, EGFR^WT^, and B-RAF^WT^, as shown in Figure 7 and Table 8.

**Table 8.** Interactions of ligands with amino acids of estrogen receptor (PDB: ID 3ERT).

No.	Protein	Ligand	Residue (H-Bond)	Length	Residue (Bond type)	Length	No. of H-Bonds	No. of total bonds	Affinity kcal mol^−1^
1	**Estrogen Receptor (PDB:ID 3ERT)**	**5d**	Glu232 (H-Bond)	2.14	Met357, (Pi-alkyl) Phe445, (sulfur)	5.21 5.51	**1**	**3**	**−8.50**
2		**5e**	Asp351 (H-Bond)	2.79	Leu391, (Pi-alkyl) Leu525, (Pi-alkyl) Met421, (Pi-alky) Ile424, (Pi-alkyl) Leu387, (Pi-alkyl) Leu536, (Pi-alkyl) Val534, (Pi-alkyl) Leu539, (Pi-alkyl) Met388, (Pi-Sigma) Met388, (Pi-Sigma) Met343, (sulfur) Lys531, (Pi-cation)	4.68 4.31 5.23 5.43 5.16 3.59 4.55 4.99 3.72 3.92 5.11 4.82	**1**	**13**	**−8.90**
3		**11a**	Lys449 (H- Bond)	2.67	Pro325, (Pi-alkyl) Arg394, (Pi-alkyl) Met357, (Pi-alkyl) Glu323, (Pi- cation) Ile326, (Pi-Sigma) Ile326, (Pi-Sigma) Met343, (sulfur) Phe445,(Amid-Pi-stack)	5.06 5.03 4.48 3.98 3.99 3.64 3.88 5.57	**1**	**9**	**−8.40**
4		**11b**	Lys449 (H- Bond)	2.99	Pro325, (Pi-alkyl) Met357, (Pi-alkyl) Arg394, (Pi-alkyl) Glu323, (Pi- cation) Ile326, (Pi-Sigma) Ile326, (Pi-Sigma) Arg394, (Pi-cation)	5.38 4.50 5.12 4.30 3.47 2.98 4.80	**1**	**8**	**−8.90**
5		**5-fluorouracil**	Glu353 (H-Bond)	2.15	Ile386, (halogen) Met357, (Pi-alkyl) Pro324, (Pi-alkyl) Glu353, (Pi- cation)	3.44 5.16 4.75 4.89 4.83	**1**	**5**	**−6.4**

#### Docking and interaction with vascular endothelial growth factor receptors

**Figure 8.**
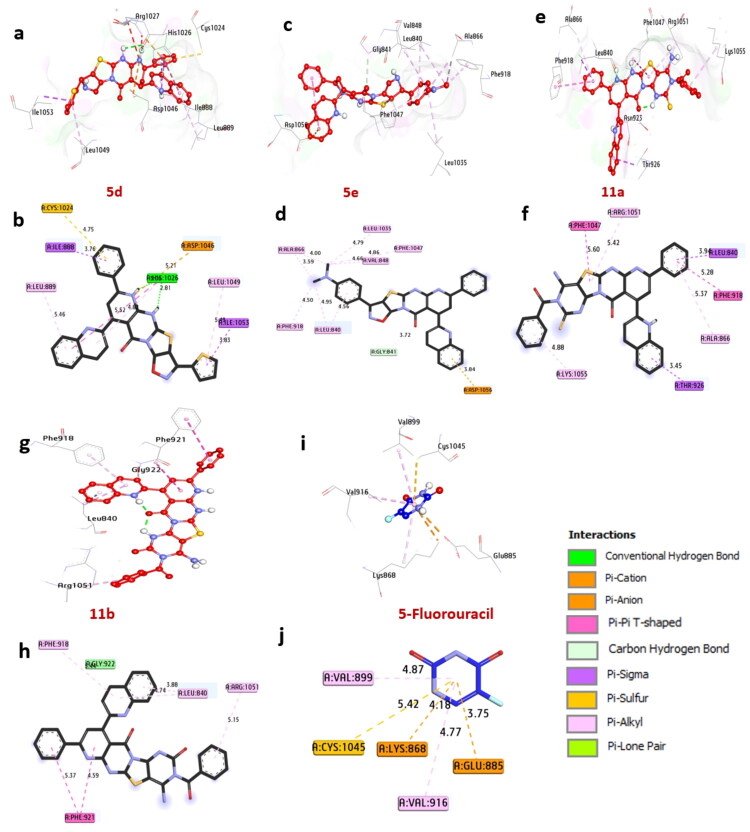
3D Representations of compounds at the binding pocket of vascular endothelial growth factor receptors (VEGFR) (PDB:ID 3WZE): (A and B) **5d**, (C and D) **5e**, (E and F) **11a,** (G and H) **11b**, and (I and J) 5-fluorouracil.

Vascular endothelial growth factor receptors (VEGFR) are a family of receptor tyrosine kinases that play a crucial role in angiogenesis, forming new blood vessels. These receptors are important targets in cancer therapy, as tumours often promote angiogenesis to ensure a sufficient blood supply for their growth. Docking results indicate that compounds 5d, 5e, 11a, and 11b exhibit strong binding affinities, with binding energies of −10.90, −10.70, −10.30, and −10.70 kcal/mol, respectively. In comparison, 5-fluorouracil has a lower binding energy of −8.50 kcal/mol. Only compound 5d forms hydrogen bonds with the key amino acid His1026. Additionally, several non-hydrophilic bond interactions were identified, including alkyl bonds with the following residues: Leu1049, His1026, Leu889, Leu1035, Phe1047, Val848, Ala866, Phe918, Leu840, Arg1051, and Lys1055. There were also pi-sigma interactions with Thr926, Leu840, Ile1053, and Ile888 and amide-pi stacking interactions with Phe918, Phe1047, and Phe921. Other notable interactions included carbon-hydrogen bonds with Gly841, pi-cation interactions with Asp1056 and Asp1046, and pi-sulfur interactions with Cys1024. In the catalytic site, the residues His1026, Phe918, Ile888, and Leu840 were found to influence the binding affinity of the compounds positively. These findings indicate that compounds **5d**, **5e**, **11a**, and **11b** are the most promising candidates for further investigation as potential vascular endothelial growth factor receptor (VEGFR-2) inhibitors. This aligns with studies conducted by (El-Khouly et al. 2022; Elmorsy et al. 2022; Khaled et al. 2023), which also assessed compounds with favorable binding properties using in-silico molecular docking simulations against VEGFR-2, EGFR^WT^, and B-RAF^WT^, as illustrated in Figure 8 and detailed in Table 9.

**Table 9.** Interactions of ligands with amino acids of VEGFR (PDB:ID 3WZE).

No.	Protein	Ligand	Hydrophilic Interactions		Hydrophobic Contacts		No. of H-Bonds	No. of total bonds	Affinity kcal mol^−1^
			Residue (H-Bond)	Length	Residue (Bond type)	Length			
**1**	**VEGFR (PDB:ID 3WZE)**	**5d**	His1026 (H-Bond)	2.81	Leu1049, (Pi-alkyl) His1026, (Pi-alkyl) Leu889, (Pi-alkyl) Ile1053, (Pi-sigma) Ile888, (Pi-sigma) Cys1024, (Pi-sulfur) Asp1046, (Pi-cation)	5.49 4.88 5.46 3.83 3.76 4.75 5.21	1	8	−10.90
**2**		**5e**	–	–	Leu1035, (Pi-alkyl) Phe1047, (Pi-alkyl) Val848, (Pi-alkyl) Ala866, (Pi-alkyl) Phe918, (Pi-alkyl) Leu840, (Pi-alkyl) Gly841, (Carbon H bond) Asp1056, (Pi-cation)	4.79 4.86 4.66 4.00 3.59 4.50 3.72 3.84	0	8	−1070
**3**		**11a**	–	–	Arg1051, (Pi-alkyl) Ala866, (Pi-alkyl) Lys1055, (Pi-alkyl) Thr926, (Pi-Sigma) Leu840, (Pi-Sigma) Phe918, (Amide pi stacked) Phe1047,(Amide pi stacked)	5.42 5.37 4.88 3.45 3.99 5.28 5.60	0	7	−10.30
**4**		**11b**	–	–	Arg1051, (Pi-alkyl) Leu840, (Pi-alkyl) Phe918, (Pi-alkyl) Phe921, (Amide pi stacked) Phe921,(Amide pi stacked)	5.15 4.74 3.44 4.59 5.37	0	5	−10.70
**5**		**5-fluorouracil**	–	–	Cys1045, (sulfur) Val916, (Pi-alkyl) Val899, (Pi-alkyl) Glu885, (Pi- cation) Lys868, (Pi- cation)	5.42 4.77 4.87 3.75 4.18	0	5	−8.50

#### In silico pharmacokinetics ADME prediction of synthesized compounds

**Figure 9.**
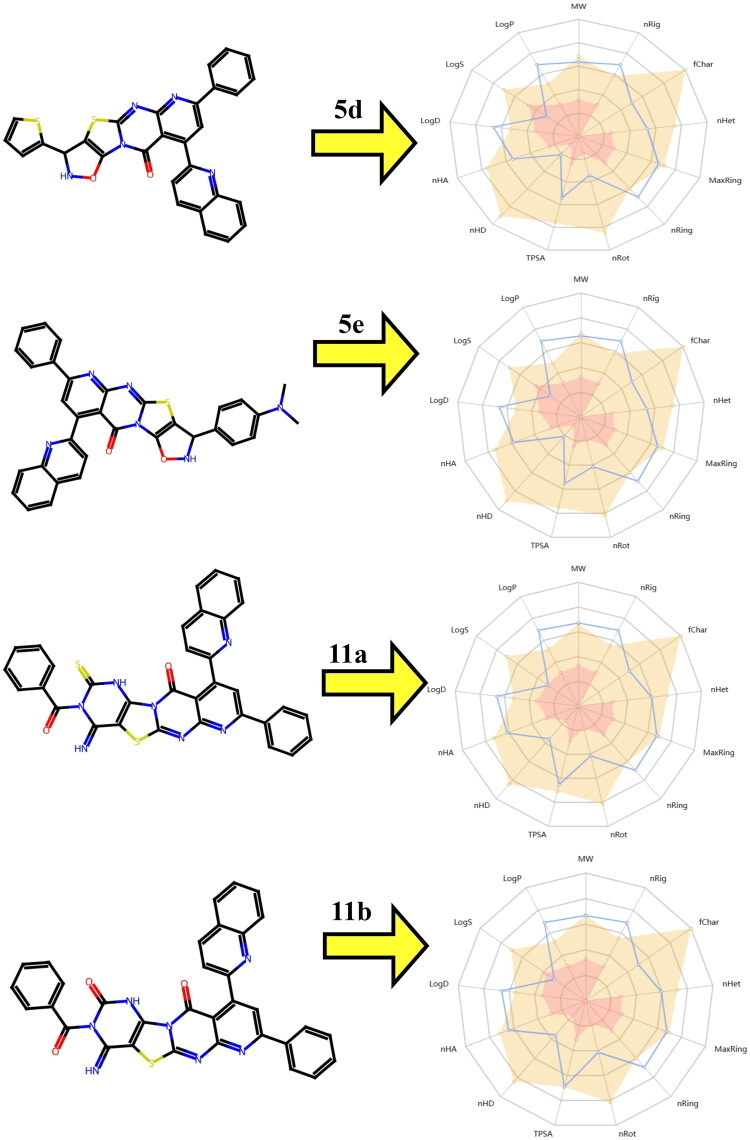
Oral bioavailability graph for compounds.

Based on the molecular docking results, the compounds **5d**, **5e**, **11a**, and **11b** have been identified as the most promising candidates due to their high affinity related to ADME (Absorption, Distribution, Metabolism, and Excretion) and their toxicity risks. Firstly, the physicochemical properties of the tested compounds are presented in Table 10 and Figure 9. All relevant physiochemical criteria were thoroughly investigated and assessed. Each compound has an adequate number of rotatable bonds (RBs), with a count of 4, which is crucial for structural flexibility. Compounds with fewer than 10 RBs are more likely to be bioavailable. As RBs increase, their role in facilitating effective interactions with specific binding sites becomes even more significant. All four compounds feature fewer than 10 hydrogen bond acceptors (HBA) and fewer than 5 hydrogen bond donors (HBD), indicating a balanced structure enhancing their oral bioavailability potential. Additionally, the compounds’ total polar surface area (TPSA) values are relatively modest, primarily falling within the ideal range of 60–140. This range is conducive to effective gut absorption and oral bioavailability. Next, we assessed the lipophilicity and water solubility of the compounds. The results show that all active compounds are slightly soluble in water, with Log S values ranging from −7.261 to −4.167 for compounds **5d**, **5e**, **11a**, and **11b**, indicating moderate water solubility. Soluble molecules facilitate the synthesis, handling, and formulation of bioactive substances. Furthermore, pharmacokinetic tests were conducted on the compounds.

**Table 10.** Prediction of pharmacokinetics and physicochemical properties of compounds.

Id	ID	5d	5e	11a	11b	Id	ID	5d	5e	11a	11b
Physicochemical Properties	MW	531.08	568.17	609.1	593.13	Metabolism	CYP1A2-inh	0.513	0.266	0.341	0.312
	Vol	509.087	568.123	589.719	580		CYP1A2-sub	0.131	0.144	0.138	0.137
	Dense	1.043	1	1.033	1.023		CYP2C19-inh	0.591	0.503	0.576	0.596
	nHA	7	8	9	10		CYP2C19-sub	0.064	0.067	0.054	0.052
	nHD	1	1	2	2		CYP2C9-inh	0.477	0.457	0.592	0.483
	TPSA	81.41	84.65	121.79	138.86		CYP2C9-sub	0.693	0.711	0.142	0.052
	nRot	3	4	4	4		CYP2D6-inh	0.002	0.015	0.561	0.797
	nRing	8	8	8	8		CYP2D6-sub	0.461	0.764	0.177	0.092
	MaxRing	16	16	17	17		CYP3A4-inh	0.297	0.279	0.47	0.388
	nHet	9	9	11	11		CYP3A4-sub	0.718	0.755	0.185	0.174
	fear	0	0	0	0	Excretion	CL (Clearance)	3.187	4.127	5.075	3.488
	nRig	42	43	47	47		T12	0.033	0.04	0.249	0.287
	Flex	0.071	0.093	0.085	0.085	Toxicity	hERG Blockers	0.01	0.064	0.032	0.094
	nStereo	1	1	0	0		H-HT	0.853	0.9	0.951	0.946
Solubility	LogS	−7.261	−6.52	−4.167	−5.119		DILI	0.951	0.938	0.992	0.987
	LogD	4.423	4.431	4.432	4.017		AMES Toxicity	0.765	0.925	0.309	0.149
	LogP	5.774	6.226	5.382	5.282		Rat OralToxicity	0.051	0.062	0.057	0.015
	ESOL Log S	−7.00	−7.37	−7.32	−7.72		FDAMDD	0.973	0.97	0.963	0.886
	Ali Log S	−8.04	−7.94	−9.76	−8.82		Skin Sensitization	0.019	0.023	0.027	0.025
	Silicon-IT class	Poorly	Poorly	Poorly	Poorly		Carcinogenicity	0.778	0.866	0.988	0.977
drug-likeness	**Lipinski Rule**	Rejected	Rejected	Rejected	Rejected		Eye Corrosion	0.003	0.003	0.003	0.003
	**Pfizer Rule**	Accepted	Accepted	Accepted	Accepted		Eye Irritation	0.016	0.02	0.012	0.021
	**Golden Triangle**	Rejected	Rejected	Rejected	Rejected		Respiratory Toxicity	0.046	0.072	0.047	0.044
Absorption	Pgp-inh	0.997	1	0.999	0.904	Toxicophoric Rules	Non-Genotoxic Carcinogenicity	1	2	1	1
	Pgp-sub	0.148	0.983	0.001	0.039		LD50_oral	1	1	1	1
	HIA	0.004	0.008	0.007	0.011		Genotoxic Carcinogenicity	0	0	0	0
	F (20%)	0.929	0.013	0.022	0.526		Sure ChEMBL	0	0	0	0
	F (30%)	0.193	0.4	0.002	0.042		Non-Biodegradable	0	0	1	1
	Caco-2	−4.937	−4.891	−5.085	−5.084		Skin Sensitization	0	0	0	0
	MDCK	1.40E-05	1.39E-05	1.61E-05	2.04E-05		Aquatic Toxicity	1	2	1	1
Distribution	BBB	0.036	0.033	0.011	0.007	Medicinal Chemistry	Toxicophores	3	2	4	2
	PPB%	100.09%	98.87%	99.73%	98.94%		QED	0.291	0.269	0.225	0.296
	VDss	0.532	0.471	0.125	0.03		Synth	3.692	3.687	3.274	3.236
	Fu %	1.33%	1.41%	0.93%	0.82%		Fsp3	0.034	0.091	0	0

The results indicate that the compounds studied have high theoretical bioavailability and may be potential drug candidates. However, all compounds exhibited moderate intestinal absorption and did not interact with other medications, as they do not inhibit the CYP2C9, CYP2C19, or CYP1A2 enzymes. The study also assessed the drug-likeness of the compounds using the Lipinski, Golden Triangle, and Pfizer criteria. While all compounds met the drug-likeness criteria of the Pfizer rules, they did not fully comply with the requirements of the Lipinski and Golden Triangle rules. This suggests that they possess favorable physicochemical properties for drug development.

The distribution of the compounds, including plasma protein binding (PPB), was thoroughly examined. All compounds demonstrated over 98% binding to plasma proteins, indicating high levels of protein-bound plasma, a narrow therapeutic index, and a low fraction of unbound plasma. However, the penetration of these compounds across the blood-brain barrier (BBB) was poor, meaning they cannot cross the BBB. Lastly, computational analysis suggests that compounds **5d**, **5e**, **11a**, and **11b** are relatively safe and non-toxic, as indicated by the findings in Table 11.

**Table 11.** Prediction of toxicity risks and oral toxicity prediction results of compounds.

No.	Ligand	Toxicity risks				Physicochemical properties					
		Mutagenic	Tumorigenic	Irritant	Reproductive	CLogP	Solubility	Molecular Weight	TPSA	Drug likeness	Drug score
1	**5d**	(−)	(−)	(−)	(−)	5.88	−9.55	531.0	133.2	3.77	0.23
2	**5e**	(−)	(+)	(−)	(−)	5.91	−9.58	568.0	108.2	2.48	0.12
3	**11a**	(−)	(−)	(−)	(−)	5.61	−9.92	609.0	174.5	3.19	0.20
4	**11b**	(−)	(−)	(−)	(−)	5.25	−9.84	593.0	159.5	3.52	0.22

#### Molecular dynamics simulation (MDS)

**Figure 10.**
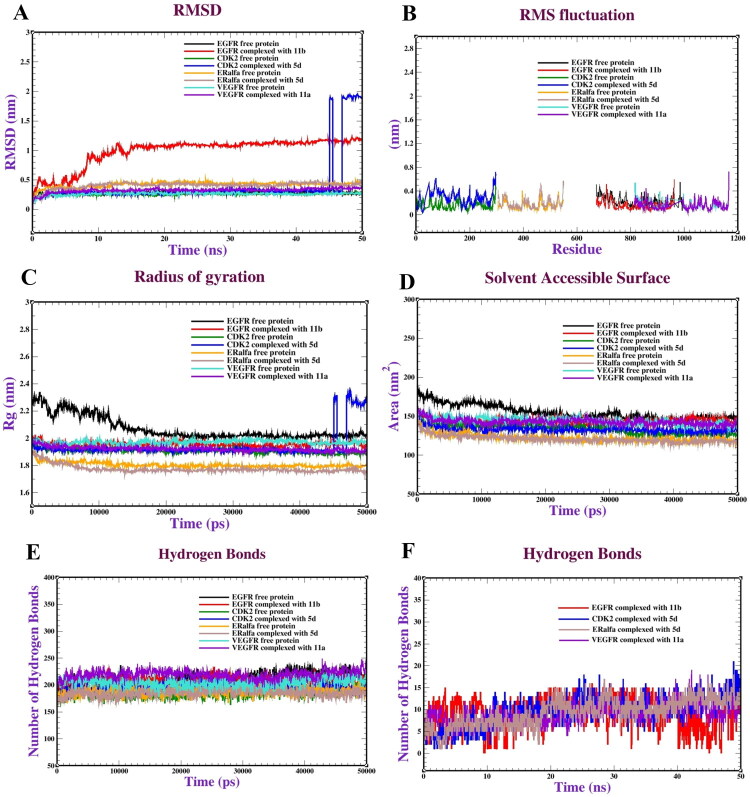
Molecular dynamics of EGFR (PDB: ID 1M17), CDK2 (PDB: ID 2A4L), ERα (PDB: ID 3ERT), and VEGFR (PDB: ID 3WZE) complexed with **11b**, **5d**, and **11a**: (A) RMSD, (B) RMSF, (C) SASA, (D) Radius of gyration (Rg), (E) Intermolecular hydrogen bonds and (F) Intramolecular hydrogen bonds.

After analyzing the interactions of four anticancer protein receptors with the compounds 11b, 5d, and 11a, we conducted dynamic simulations to investigate the behavior and stability of the protein complexes at the atomic level. Initially, we performed multiple Molecular Dynamics Simulations (MDS) on the following proteins: EGFR (PDB ID: 1M17), CDK2 (PDB ID: 2A4L), ERα (PDB ID: 3ERT), and VEGFR (PDB ID: 3WZE), each complexed with the compounds **11b**, **5d**, and **11a**. This was done to evaluate their stability and dynamics. We assessed the stability of the protein structures using the Root Mean Square Deviation (RMSD) method, as shown in Figure 10(A) The RMSD values for the CDK2, ERα, and VEGFR proteins complexed with compounds **5d** and **11a** showed stability, ranging from 0.22 to 0.24 nm for CDK2, 0.30–0.35 nm for ERα, and 0.18–0.20 nm for VEGFR. These values stabilized after 10, 15, and 20 nanoseconds, respectively. In contrast, the RMSD values for the EGFR protein complexed with compound **11b** were also stable, ranging from 0.30 to 1.00 nm, with stabilization occurring after 25 nanoseconds. We also evaluated the flexibility of the amino acid residues during the simulation using Root Mean Square Fluctuation (RMSF) analysis. This analysis demonstrated minimal variability, as most residues exhibited small fluctuations (0.1–0.6 nm), indicating relative stability for the EGFR, CDK2, ERα, and VEGFR protein receptors see Figure 10(B). Following this, we performed a Radius of Gyration (Rg) analysis to assess the overall shape of the protein complexes. The Rg values reflect the compactness or expansion of the protein structures during the simulation. For the complexes involving CDK2 and VEGFR with **5d** and **11a**, the Rg values ranged from 1.85 to 1.90 nm and 1.80 to 1.90 nm, respectively, as shown in Figure 10(C). The Rg values for EGFR and ERα with **11b** and **5d** were also stable, ranging from 2.00 to 2.20 nm and 1.75 to 1.80 nm, respectively. Additionally, we utilized the Solvent Accessible Surface Area (SASA) to evaluate the exposure of the proteins to surrounding solvent molecules. The SASA values for CDK2 and VEGFR with **5d** and **11a** ranged from 135 to 145 nm^2^ and 155 to 165 nm^2^, respectively. Similarly, the SASA values for EGFR and ERα with **11b** and **5d** remained stable, ranging from 160 to 175 nm^2^ and 125 to 130 nm^2^, respectively, as shown in Figure 10(D). We observed that intramolecular hydrogen bonds play a crucial role in determining the characteristics of the complexes, including their structure, stability, and reactivity. As illustrated in Figure 10(E), the complexes involving the CDK2 and ERα receptors with compound 5d exhibit bond strengths ranging from 170 to 230. Similarly, the EGFR and VEGFR proteins with compounds **11b** and **11a** display bond strengths between 210 and 220 and 240–250, respectively. In terms of intermolecular hydrogen bonds, most protein-receptor complexes showed a significant number of interactions, ranging from 2 to 12 bonds. These interactions are essential for enhancing the stability of the intricate structures formed by these complexes, as shown in Figure 10(F). These findings are consistent with studies (Al-Rashood et al. 2020; El-Khouly et al. 2022; Sroor et al. 2024; Bawazir et al. 2025), which also performed Molecular Dynamics (MD) simulations to examine compounds’ stability and molecular interactions with VEGFR-2, EGFR^WT^, CDK2, and ERα. These studies used similar metrics, including RMSD, RMSF, SASA, and hydrogen bonds, to evaluate their results.

## Conclusions

In this study, we synthesized new substituted quinoline derivatives, including pyrido[2,3-*d*] thiazolo[3,2-*a*] pyrimidinones, isoxazolothiazolopyrido[2,3-*d*] pyrimidinones, pyridopyrimido-thiazolo[4,5-*d*] [1,3] thiazinones, and pyrimido [4′,5′:4, 5] thiazolo[2,3-*b*] pyridopyrimidinones. We achieved high yields in synthesizing these compounds using recent methods and advanced techniques. We tested the activity of all synthesized compounds at the minimum inhibitory concentration (MIC) against several cancer cell lines, including human oral carcinoma cells (KB), human nasopharyngeal carcinoma cells (CNE2), human gastric carcinoma cells, and human breast carcinoma cells (MCF-7). Most of these compounds demonstrated good activity compared to the standard drug, fluorouracil. Among them, the pyrimido-thiazolo[2,3-*b*] pyridopyrimidinones and isoxazolo-thiazolo[3,2-a] pyrido [2,3-*d*] pyrimidinones exhibited the best activity against multiple cancer cell lines, likely due to the presence of isoxazole, thiazole, pyrimidine, pyridine, and quinoline rings, along with various functional groups such as amino, thioxo, carbonyl, carbonitrile, NH, and methyl groups. Compounds **5d**, **5e**, **11a**, and **11b** demonstrated superior activity to the reference drug 5-fluorouracil. Docking simulations identified compounds **5d**, **5e**, **11a**, and **11b** as promising anticancer agents due to their intense binding energies and interactions with key receptors (EGFR, CDK2, ERα, VEGFR), mediated by diverse molecular forces. These compounds also exhibited favorable in-silico ADMET profiles, aligning with Pfizer’s rules for drug-likeness, indicating good oral absorption, low toxicity, and optimal physicochemical properties. Molecular dynamics (MD) simulations further validated the stability of complexes formed by **5d**, **11a**, and **11b** with the receptors. Key stability metrics included low RMSD values (0.18–1.00 nm), moderate RMSF fluctuations (0.10–0.6 nm), consistent solvent-accessible surface area (SASA) values (125–175 nm^2^), and stable radius of gyration (Rg) values (1.75–2.20 nm), confirming structural integrity during simulations. Their robust receptor binding, favorable pharmacokinetic profiles, and stable molecular dynamics behavior suggest they could effectively inhibit cancer-related enzymes while minimizing toxicity risks.

## Supplementary Material

SUPPLEMENTARY MATERIAL_A A ABU_HASHEM.doc

## Data Availability

All data are contained within the article.
