# Supplementary material for: Design, evaluation, cytotoxic activity, molecular docking, ADMET analysis, and dynamic simulations and the preparation of new isoxazoles, thiazoles, 1,3-thiazines, and thiazolopyrimidines derived from quinoline-pyridopyrimidines
Source: Pharm Biol. 2025 Aug 19;63(1):607–44. doi: 10.1080/13880209.2025.2547744 (PMC12366517; doi:10.1080/13880209.2025.2547744)
Supplement: SUPPLEMENTARY MATERIAL_A A ABU_HASHEM.doc [file IPHB_A_2547744_SM9566.doc]

**Supplementary Material Data**

**Design, Evaluation, Cytotoxic Activity, Molecular docking, ADMET analysis, and Dynamic Simulations and The Preparation of New Isoxazoles, Thiazoles, 1,3-Thiazines, and Thiazolopyrimidines Derived from Quinoline-Pyridopyrimidines**

Ameen A. Abu-Hashem ^a^, ^b^, *, Nasser Amri ^a^, Ahmed F. El-Sayed ^c, d^

^a^ Department of Physical Sciences, Chemistry Division, College of Science, Jazan University, Jazan 45142, Saudi Arabia; ^b^ Photochemistry Department (Heterocyclic Unit), National Research Centre, 12622 Dokki, Giza, Egypt; ^c^ Microbial Genetics Department, Biotechnology Research Institute, National Research Centre, Giza, Egypt; ^d^ Egypt Center for Research and Regenerative Medicine (ECRRM), Cairo, Egypt.

***** Correspondence: [aminaliabuhashem@yahoo.com](mailto:aminaliabuhashem@yahoo.com) or [aaabuhashem@jazanu.edu.sa](mailto:aaabuhashem@jazanu.edu.sa); Tel.: +966-0591363915; +2-01225211700 (A. A. Abu-Hashem)

| **Table of Contents** | **Page** |
| --- | --- |
| Figure S1 ^1^H NMR Spectrum (500 MHz, DMSO-*d6*) of Compound **2** | 2 |
| Figure S2 ^1^H NMR Spectrum (500 MHz, DMSO-*d6*) of Compound **3a** | 3 |
| Figure S3 ^13^C NMR Spectrum (125 MHz, DMSO-*d6*) of Compound **3a** | 4 |
| Figure S4 ^1^H NMR Spectrum (500 MHz, DMSO-*d6*) of Compound **5a** | 5 |
| Figure S5 ^13^C NMR Spectrum (125 MHz, DMSO-*d6*) of Compound **5a** | 6 |
| Figure S6 ^1^H NMR Spectrum (500 MHz, DMSO-*d6*) of Compound **6** | 7 |
| Figure S7 ^13^C NMR Spectrum (125 MHz, DMSO-*d6*) of Compound **6** | 8 |
| Figure S8 ^1^H NMR Spectrum (500 MHz, DMSO-*d6*) of Compound **7a** | 9 |
| Figure S9 ^13^C NMR Spectrum (125 MHz, DMSO-*d6*) of Compound **7a** | 10 |
| Figure S10 ^1^H NMR Spectrum (500 MHz, DMSO-*d6*) of Compound **8** | 11 |
| Figure S11 ^1^H NMR Spectrum (500 MHz, DMSO-*d6*) of Compound **9** | 12 |
| Figure S12 ^1^H NMR Spectrum (500 MHz, DMSO-*d6*) of Compound **10a** | 13 |
| Figure S13 ^13^C NMR Spectrum (125 MHz, DMSO-*d6*) of Compound **10a** | 14 |
| Figure S14 ^1^H NMR Spectrum (500 MHz, DMSO-*d6*) of Compound **11a** | 15 |
| Figure S15 ^13^C NMR Spectrum (125 MHz, DMSO-*d6*) of Compound **11a** | 16 |

**^1^H- NMR and C^13^** **- NMR spectra**

***8-phenyl-6-(quinolin-2-yl)-5H-pyrido[2,3-d]thiazolo[3,2-a]pyrimidine-3, 5(2H)-dione* (2)**


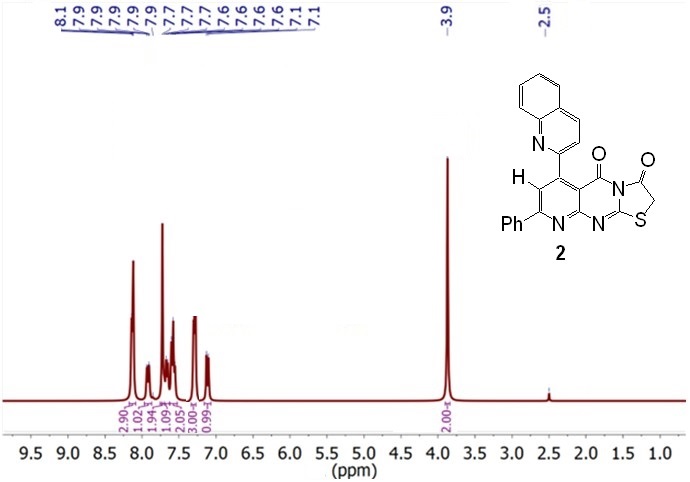


Figure S1 ^1^H NMR Spectrum (500 MHz, DMSO-*d6*) of Compound **2**

***2-benzylidene-8-phenyl-6-(quinolin-2-yl)-5H-pyrido[2,3-d] thiazolo[3,2-a]pyrimidine-3,5(2H)-dione* (3a)**


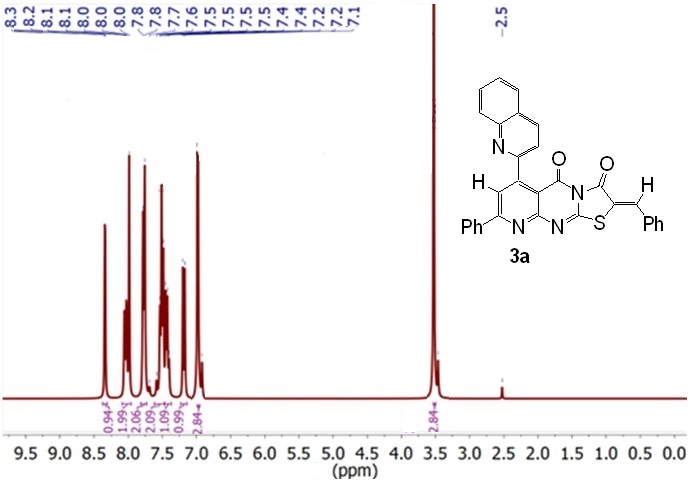


Figure S2 ^1^H NMR Spectrum (500 MHz, DMSO-*d6*) of Compound **3a**

***2-benzylidene-8-phenyl-6-(quinolin-2-yl)-5H-pyrido[2,3-d] thiazolo[3,2-a]pyrimidine-3,5(2H)-dione* (3a)**


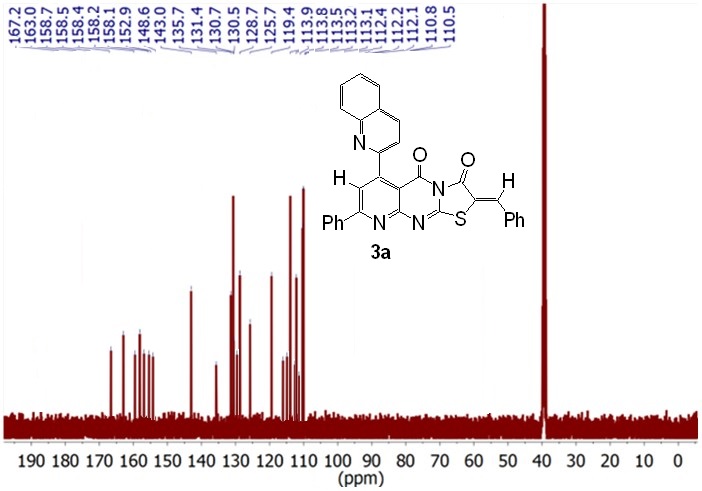


Figure S3 ^13^C NMR Spectrum (125 MHz, DMSO-*d6*) of Compound **3a**

***3,7-diphenyl-9-(quinolin-2-yl)-2,3-dihydro-10H-isoxazolo[5',4':4,5]thiazolo[3,2-a]pyrido[2,3-d] pyrimidin-10-one* (5a)**


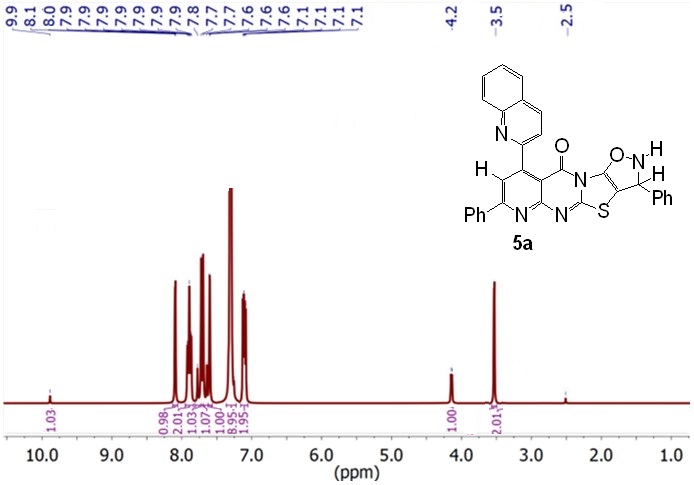


Figure S4 ^1^H NMR Spectrum (500 MHz, DMSO-*d6*) of Compound **5a**

***3,7-diphenyl-9-(quinolin-2-yl)-2,3-dihydro-10H-isoxazolo[5',4':4,5]thiazolo[3,2-a]pyrido[2,3-d] pyrimidin-10-one*(5a)**


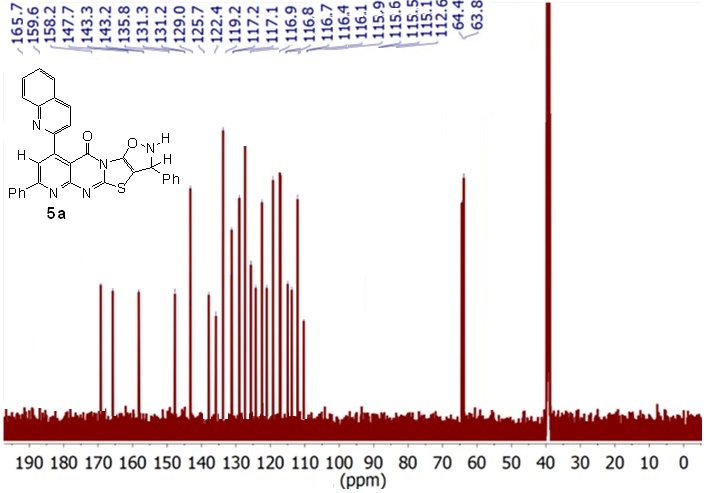


Figure S5 ^13^C NMR Spectrum (125 MHz, DMSO-*d6*) of Compound **5a**

***3-amino-5-oxo-8-phenyl-******6-(quinolin-2-yl)-5H-pyrido[2,3-d]thiazolo[3,2-a]pyrimidine -2-carbonitrile* (6)**


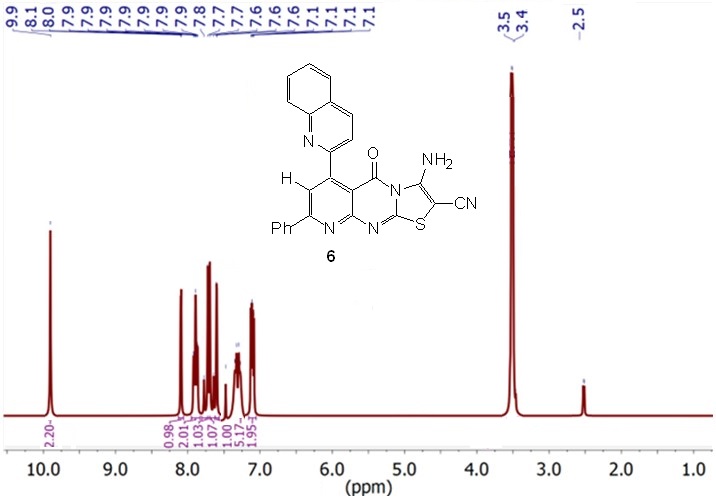


Figure S6 ^1^H NMR Spectrum (500 MHz, DMSO-*d6*) of Compound **6**

***3-amino-5-oxo-8-phenyl-6-(quinolin-2-yl)-5H-pyrido[2,3-d]thiazolo[3,2-a]pyrimidine -2-carbonitrile* (6)**


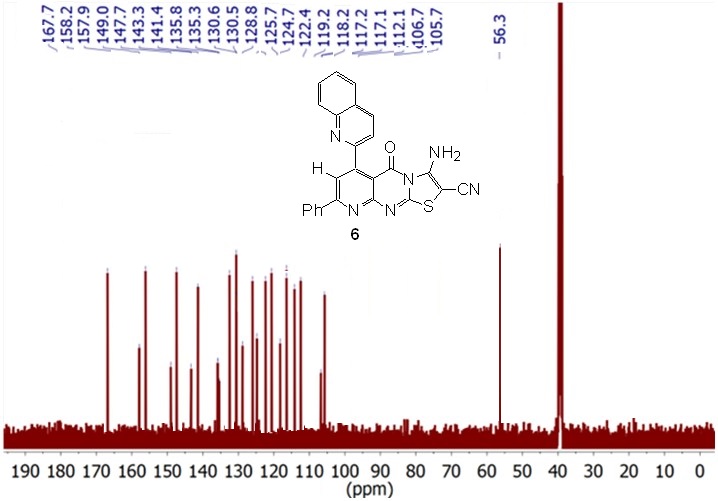


Figure S7 ^13^C NMR Spectrum (125 MHz, DMSO-*d6*) of Compound **6**

***8-phenyl-10-(quinolin-2-yl)-pyrimido[4',5':4,5] thiazolo [2,3-b]pyrido[3``,2``-e] pyrimidine-4,11(3H)-dione* (7a)**


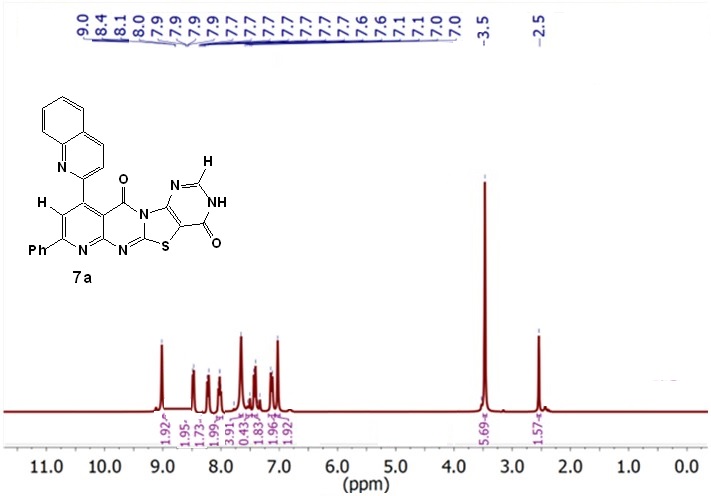


Figure S8 ^1^H NMR Spectrum (500 MHz, DMSO-*d6*) of Compound **7a**

***8-phenyl-10-(quinolin-2-yl)-pyrimido[4',5':4,5] thiazolo [2,3-b]pyrido[3``,2``-e] pyrimidine-4,11(3H)-dione* (7a)**


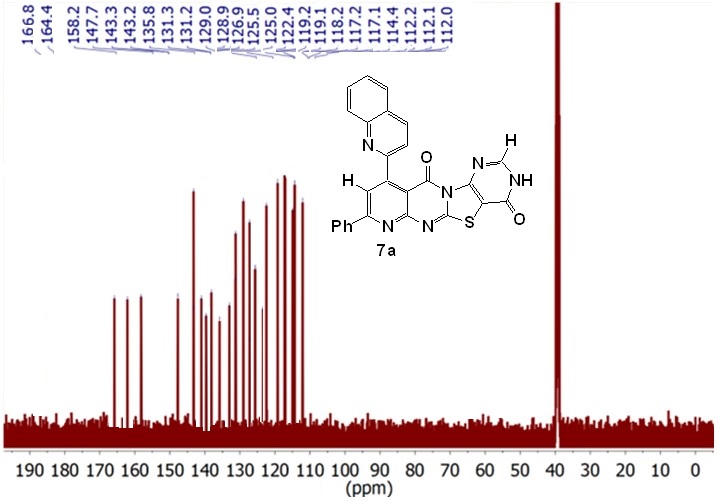


Figure S9 ^13^C NMR Spectrum (125 MHz, DMSO-*d6*) of Compound **7a**

***4-amino-8-phenyl-10-(quinolin-2-yl)-pyrimido[4',5':4,5] thiazolo [2,3-b]pyrido[3``,2``-e] pyrimidine-11-one* (8)**


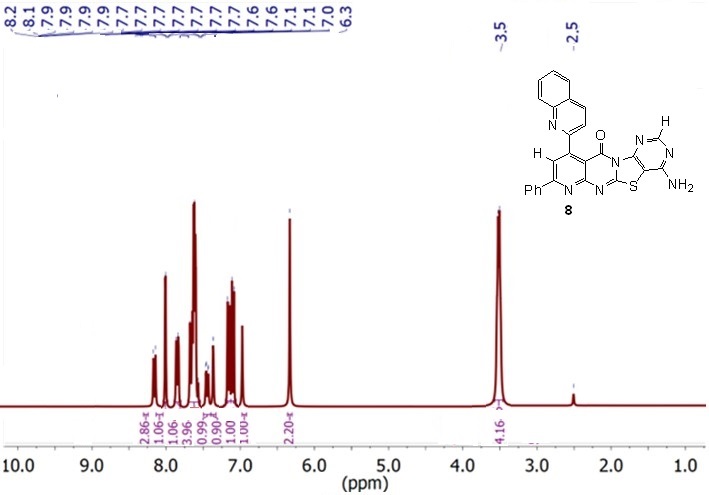


Figure S10 ^1^H NMR Spectrum (500 MHz, DMSO-*d6*) of Compound **8**

***4-imino-8-phenyl-10-(quinolin-2-yl)-2-thioxo-1,4-dihydro-2H,11H-pyrido [2'',3'':4',5'] pyrimido[2',1':2, 3]thiazolo[4,5-d][1,3]thiazin-11-one* (9)**


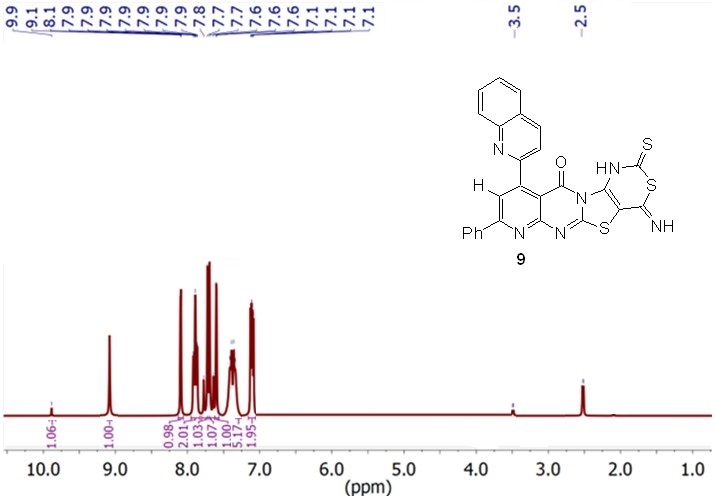


Figure S11 ^1^H NMR Spectrum (500 MHz, DMSO-*d6*) of Compound **9**

***N-((2-cyano-5-oxo-8-phenyl-6-(quinolin-2-yl)-5H-pyrido[2,3-d]*** ***thiazolo [3,2-a] pyrimidin-3-yl) carbamothioyl) benzamide* (10a)**


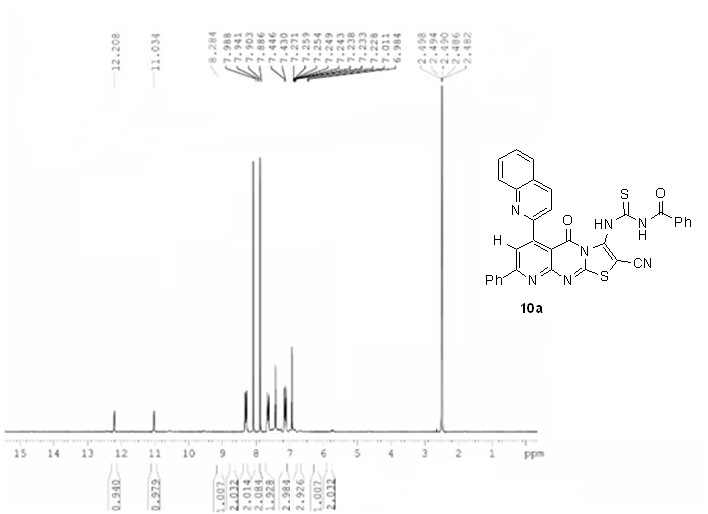


Figure S12 ^1^H NMR Spectrum (500 MHz, DMSO-*d6*) of Compound **10a**

***N-((2-cyano-5-oxo-8-phenyl-6-(quinolin-2-yl)-5H-pyrido[2,3-d] thiazolo [3,2-a] pyrimidin-3-yl) carbamothioyl) benzamide* (10a)**


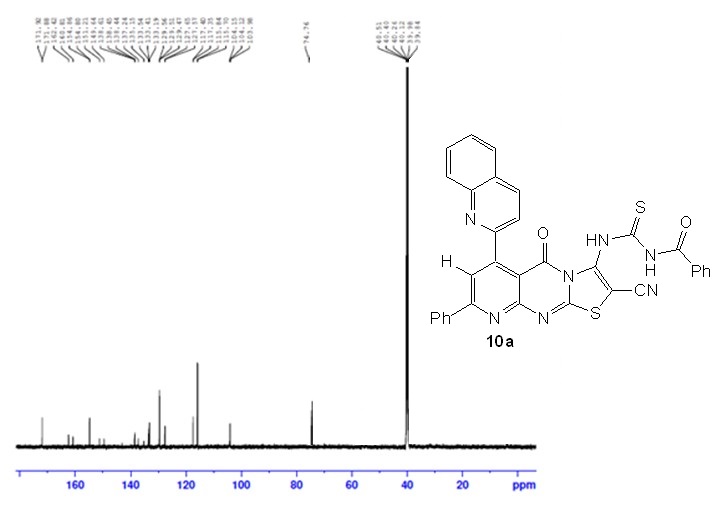


Figure S13 ^13^C NMR Spectrum (125 MHz, DMSO-*d6*) of Compound **10a**

***4-amino-3-benzoyl-2-thioxo-8-phenyl-10-(quinolin-2-yl)-pyrimido [4',5':4,5] thiazolo [2,3-b] pyrido[3``,2``-e] pyrimidine-11-one* (11a)**


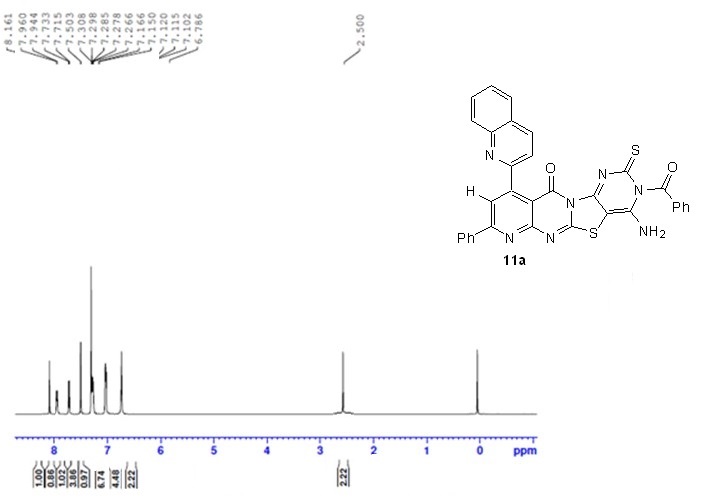


Figure S14 ^1^H NMR Spectrum (500 MHz, DMSO-*d6*) of Compound **11a**

***4-amino-3-benzoyl-2-thioxo-8-phenyl-10-(quinolin-2-yl)-pyrimido [4',5':4,5] thiazolo [2,3-b] pyrido[3``,2``-e] pyrimidine-11-one* (11a)**


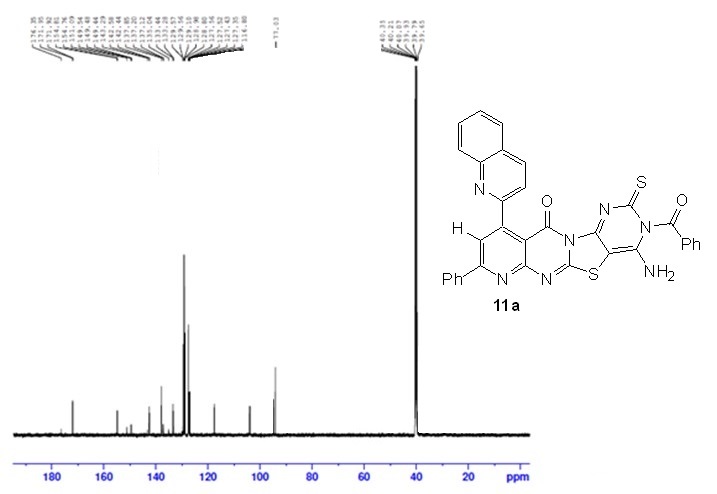


Figure S15 ^13^C NMR Spectrum (125 MHz, DMSO-*d6*) of Compound **11a**
